# Dual roles and therapeutic targeting of tumor-associated macrophages in tumor microenvironments

**DOI:** 10.1038/s41392-025-02325-5

**Published:** 2025-08-25

**Authors:** Jiasheng Xu, Lei Ding, Jianfeng Mei, Yeting Hu, Xiangxing Kong, Siqi Dai, Tongtong Bu, Qian Xiao, Kefeng Ding

**Affiliations:** 1 Department of Gastroenterology, the Second Affiliated Hospital, School of Medicine, Zhejiang University, Hangzhou, China; 2 Department of Colorectal Surgery and Oncology (Key Laboratory of Cancer Prevention and Intervention, China National Ministry of Education, Key Laboratory of Molecular Biology in Medical Sciences, Zhejiang Province, China), The Second Affiliated Hospital of Medicine, Hangzhou, China; 3 Center for Medical Research and Innovation in Digestive System Tumors, Ministry of Education, Hangzhou, China; 4 Zhejiang Provincial Clinical Research Center for Cancer, Zhejiang Province, Hangzhou, China; 5 Cancer Center, Zhejiang University, Hangzhou, China; 6 Department of General Surgery, Lanxi People’s Hospital, the Second Affiliated Hospital of Zhejiang University School of Medicine, Jinhua, China

**Keywords:** Cancer microenvironment, Tumour immunology

## Abstract

Tumor-associated macrophages (TAMs), derived from circulating monocytes recruited to tumor sites via chemotactic signals such as C-C motif ligand 2 (CCL2) and colony-stimulating factor-1 (CSF-1), are pivotal components of the tumor microenvironment (TME). Functionally polarized into distinct subtypes, TAMs play dual roles: proinflammatory M1-type TAMs enhance antitumor immunity through the secretion of cytokines such as interleukin-12 (IL-12) and tumor necrosis factor alpha (TNF-α) and direct tumor cell cytotoxicity, whereas M2-type TAMs promote tumor progression by facilitating angiogenesis, metastasis, and immunosuppression. This polarization is dynamically regulated by different cytokines, various signaling pathways, and metabolic cues within the TME. Spatial distribution analyses revealed that M2-like TAMs predominantly infiltrate hypoxic and stromal regions, where they secrete factors such as vascular endothelial growth factor (VEGF), transforming growth factor beta (TGF-β), and matrix metalloproteinases (MMPs) to remodel the extracellular matrix and suppress immune responses via programmed death-ligand 1 (PD-L1) and arginase-1 upregulation. Crucially, TAMs interact extensively with immune cells; M2-TAMs secrete interleukin-10 (IL-10) and TGF-β to inhibit cytotoxic T lymphocytes while expanding regulatory T (Treg) cells and impairing natural killer (NK) cell function via altered antigen presentation. Conversely, M1-TAMs synergize with dendritic cells to enhance T-cell priming. Therapeutically, targeting TAMs offers promising strategies, including colony-stimulating factor-1 receptor (CSF-1R) inhibitors, CCL2 antagonists, and nanoparticle-mediated repolarization of M2-TAMs toward the M1 phenotype. Emerging genetic approaches, such as clustered regularly interspaced short palindromic repeat-CRISPR-associated protein 9 (CRISPR-Cas9) editing, aim to disrupt protumorigenic pathways in TAMs. Additionally, TAM-related biomarkers (e.g., CD206 and CD163) are being evaluated for their prognostic and predictive utility in immunotherapies. Despite progress, challenges persist owing to TAM plasticity and TME heterogeneity across cancers. This review synthesizes TAM biology, immune crosstalk, and therapeutic advancements, providing a foundation for novel oncology strategies aimed at reprogramming TAMs to overcome treatment resistance and improve clinical outcomes.

## Introduction

The tumor microenvironment (TME) is a complex ecosystem in which dynamic interactions between malignant cells, stromal components, and immune populations affect disease progression and therapeutic efficacy. Among these components, tumor-associated macrophages (TAMs) have emerged as key regulators of tumor biology and, depending on their phenotypic polarization and spatial distribution, act as a double-edged sword, both inhibiting and promoting malignancy. TAMs are a subpopulation of macrophages present in the tumor microenvironment. They originate from monocytes in the peripheral blood and move through the circulatory system to the tumor tissue, where they play important biological roles.^1^ The presence and development of TAMs in tumors are closely related to the interaction of the immune system with the tumor. The number and activity of TAMs in the tumor microenvironment are regulated by a variety of factors, including cytokines, chemical signals, and other immune cell interactions^2,3^ At the same time, their functional plasticity allows them to regulate the immune response, angiogenesis, and metastasis, making them important in cancer biology and therapeutic resistance.^4,5^

Recent studies have shown that TAMs contribute to immunosuppression by limiting the function of CD8+ T cells through collagen deposition and metabolic reprogramming. For example, in breast cancer, TAMs synthesize collagen in response to transforming growth factor beta (TGF-β) signaling, consume arginine and produce metabolites such as ornithine that impair cytotoxic T-cell activity.^3^ This mechano-metabolic interaction highlights how fibrosis and TAM-driven extracellular matrix (ECM) remodeling create an unfavorable microenvironment for antitumor immunity.^4^ In particular, metabolic reprogramming is a characteristic of TAMs. M1-like macrophages depend on glycolysis, whereas M2-like TAMs preferentially utilize oxidative phosphorylation (OXPHOS) and fatty acid oxidation (FAO).^6^ Lipid metabolism, including the processing of cholesterol and phospholipids, further determines TAM polarization and protein functions, such as the secretion of immunosuppressive cytokines.^7^ Interestingly, the glucose consumption of TAMs generally exceeds that of cancer cells, and glycolysis supports their proangiogenic and stroma remodeling activities.^6^ TAMs can also promote epithelial‒mesenchymal transformation (EMT) in cancer cells and enhance their invasion and metastasis. In pancreatic ductal adenocarcinoma (PDAC), TGF-β secreted by TAMs activates the Smad2/3/4-Snail axis, driving EMT and liver metastasis.^4^ Similarly, TAMs from cancer stem cells (CSCs) contribute to tumor heterogeneity and treatment resistance, suggesting that there is bidirectional crosstalk between CSCs and TAMs in TME maintenance.^8^

Our theoretical motivation to study TAMs stems greatly from their central role in bridging innate and adaptive immunity. TAMs interact with virtually all immune cell types, including T cells, natural killer (NK) cells, and dendritic cells (DCs), to regulate effector responses through cytokine secretion, checkpoint ligand expression, and metabolic competition. For example, M2-polarized TAMs secrete interleukin (IL)-10 and TGF-β, which inhibit cytotoxic T-cell activity while recruiting regulatory T (Treg) cells via C-C motif ligand 22 (CCL22), thereby establishing an immunosuppressive mechanism. In contrast, proinflammatory M1-like TAMs enhance antigen presentation and secrete tumor necrosis factor (TNF)-α or IL-12 to stimulate antitumor immunity. This plasticity, driven by signals such as interferon (IFN)-γ or IL-4, highlights the need to investigate the molecular pathways that govern TAM polarization. Indeed, TAMs are associated with resistance to chemotherapy, radiotherapy, and immunotherapy, making the modulation of TAMs a key strategy for improving clinical outcomes. For example, colony-stimulating factor-1 receptor (CSF-1R) inhibitors and CD47-blocking antibodies targeting TAM recruitment or phagocytosis have entered clinical trials, reflecting their translational potential.

Although there has been continuous progress in the course of TAM research, there are still several major controversies that compel us to organize, reflect and summarize systematically. The traditional M1/M2 dichotomy, although useful, oversimplifies the heterogeneity of the TAM. Single-cell RNA (scRNA-seq) sequencing has revealed distinct TAM subpopulations, such as C1Q+ macrophages in hepatocellular carcinomas and FN1+ TAMs in gliomas, which define traditional classification and exhibit unique functional characteristics. Furthermore, there is conflicting evidence regarding the prognostic value of TAMs: high infiltration of TAMs is usually associated with poor survival in patients with breast and lung cancers, but in some cases, such as those with colorectal cancer, there is a paradoxical relationship with improved prognosis. These differences may stem from spatial heterogeneity, as TAMs at the tumor margins exhibit a different phenotype than do TAMs in the tumor core, or from differences in marker selection methods. In addition, the optimal treatment strategy continues to be debated; should we eliminate TAMs completely or reprogram their polarization? Or can specific signaling axes be disrupted without exacerbating systemic immunosuppression? These questions are waiting to be discussed and resolved.

In this review, we first trace the historical milestones of TAM research, from early observations linking inflammation to cancer to modern discoveries of TAM plasticity. We then delineated the TAM subtypes, their distributions in the TME, and the clinical implications of their heterogeneity. We also dissected the signaling pathways and multilayered regulatory mechanisms that govern TAM function and explored TAM interactions with other immune cells. On the basis of these findings, we discuss the therapeutic potential of antibody‒drug conjugates (ADCs) that target TAMs and critically evaluate strategies to modulate TAMs, ranging from pharmacological interventions to gene editing. The present review organizes TAM biology within a historical and cutting-edge framework, aiming to foster innovative research and translate mechanistic insights into transformative therapies, ultimately bridging the gap between clinical discovery and clinical practice.

## TAM research history and milestones

### Origin and early discovery of TAM research

**Fig. 1:**
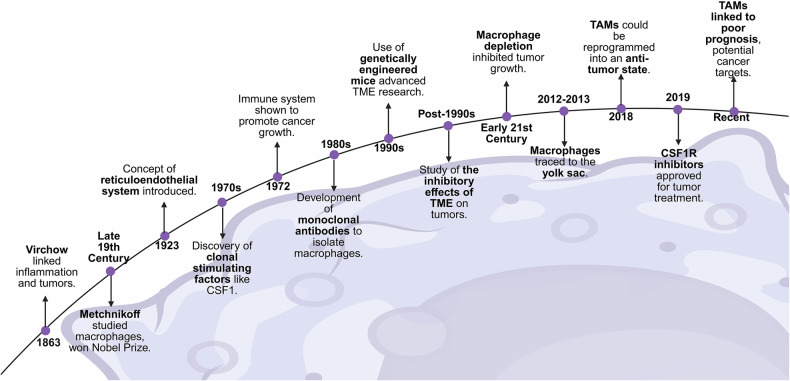
Historical advances and therapeutic insights into macrophages and tumor biology. Key milestones in macrophage and tumor biology research. Starting in 1863, Virchow linked inflammation to tumors. Metchnikoff’s macrophage studies earned a Nobel Prize (19th century), followed by the reticuloendothelial system concept (1923). In the 1970s–90s, breakthroughs in CSF1 discovery, monoclonal antibodies, and genetically engineered mice for tumor microenvironment (TME) research were reported. Later, macrophage depletion inhibited tumors (21st century), and TAMs were reprogrammed to antitumor states (2018). CSF1R inhibitors gained approval (2019), identifying TAMs as key therapeutic targets. (created with BioRender)

Since the 19th century, scientists have improved their understanding of the relationship between inflammation and cancer. In 1863, Rudolf Virchow first described the connection between inflammation and tumors, and the roles of these two cell types were previously described, suggesting that inflammation may be causally related to cancer.^9^ In the late 19th century, Elie Metchnikoff began studying phagocytic cells in the lymphatic and reticuloendothelial systems, which later became known as macrophages,^10^ and won the Nobel Prize in 1908. In 1923, the concept of the reticuloendothelial system was introduced, distinguishing macrophages from other small phagocytic cells, such as neutrophils.^11^ By the 1970s, scientists had discovered the clonal stimulating factors that induce macrophage differentiation, with colony stimulating factor-1 (CSF-1) being the first such factor discovered.^12^ In 1972, research revealed the prevailing theory that the immune system may promote cancer growth.^13^ The development of monoclonal antibody technology in the 1980s made it possible to identify and isolate macrophages. The use of genetically engineered mouse models in the 1990s further advanced cancer research. Therefore, the inhibitory effect of the TME on malignant tumors was explored after the 1990s. In the 21st century, studies have shown that the depletion of macrophages can inhibit tumor growth and metastasis while promoting angiogenesis. In 2012–2013, scientists discovered that resident macrophages in tissues originate from the yolk sac.^14^ In 2018, research confirmed that tumor-associated macrophages (TAMs) can be reprogrammed into an antitumor state. In 2019, a CSF1R inhibitor was approved for the treatment of certain types of tumors. Recent research has revealed an association between TAMs and poor prognosis, providing a new perspective for cancer treatment^15^ (Fig. 1).

### Key findings of TAM research

Tumor-associated macrophages play a pivotal role in the tumor microenvironment, as extensively studied, highlighting their contribution to tumor progression and metastasis. These cells exert immunosuppressive effects through various mechanisms and are associated with the invasion and metastasis of cancer cells. The phenotypic diversity of TAMs has been observed across different tumor types, with multiple TAM subtypes identified, some of which are linked to poor prognosis. The subtypes of TAMs associated with poor prognosis mainly include M2 TAMs. M2 TAMs promote the antiapoptotic ability of tumor cells by secreting IL-6, IL-10 and other cytokines and increase the resistance of tumor cells to chemotherapy drugs. For example, M2 TAMs in breast cancer tissue promote resistance to doxorubicin in tumor cells through the paracrine circuit of IL-6.^16^ Additionally, M2 TAMs can inhibit the activation and proliferation of cytotoxic T lymphocytes (CTLs), weaken their antitumor immunity, and reduce the antitumor effect of cytotoxic drugs.^17^Transcriptome-wide profiling of TAMs has revealed their complex roles in the tumor microenvironment, including immunosuppression and promotion of angiogenesis.^18,19^ Single-cell RNA sequencing studies have provided insights into the heterogeneity of TAMs, revealing distinct phenotypes and functions within tumors. The investigation of TAMs has propelled the consideration of macrophages as targets for cancer therapy, particularly in clinical trials targeting CD47.^20^ The origin and dynamics of TAMs have been elucidated through fate mapping techniques, shedding light on their differentiation process within the tumor microenvironment. TAMs have demonstrated immunosuppressive and proangiogenic properties in both in vitro and in vivo experiments, which are closely related to tumor growth and metastasis. The clinical importance of TAMs is underscored by their correlation with poor prognosis in various human cancers, indicating their potential significance in cancer therapy.

## Types and distribution of TAMs

### Comparison of M1-type macrophages and M2-type macrophages

#### Definitions of M1 and M2

Macrophages are white blood cells located in tissues and are derived from monocytes, which in turn are derived from precursor cells in the bone marrow. Macrophages are involved in both nonspecific and specific immunity and are immune cells with a variety of functions. M1 and M2 cells are two distinct subpopulations of macrophages and are classified according to their activation status and function. M1 macrophages are classically differentiated and activated by interferon-γ^21^ and LPS (cytoplasmic polysaccharides), whereas M2 macrophages are selectively differentiated and activated by Th2 (helper cell 2) cytokines and inflammatory factors such as IL-4 and IL-13.^22^ In terms of function, M1 macrophages secrete mainly proinflammatory factors and phagocytose pathogens and apoptotic cells, whereas M2 macrophages secrete mainly inhibitory inflammatory factors that suppress inflammatory responses and act on tissue repair and remodeling.^23^

#### Differential significance of M1- and M2-type macrophages in TAMs

**Fig. 2:**
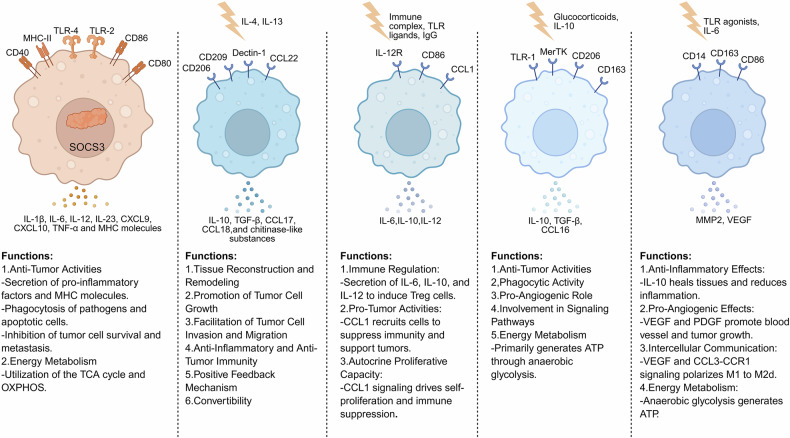
Macrophage polarization states and functional diversity in the tumor microenvironment. This figure illustrates the distinct polarization states of macrophages and their roles in the tumor microenvironment. M1 macrophages, which are activated by TLR ligands, secrete proinflammatory cytokines such as IL-6 and TNF-α, which exhibit antitumor activities and rely on OXPHOS for energy. In contrast, M2 macrophages, which are polarized by IL-4 and IL-13, promote tissue remodeling, tumor cell growth, and anti-inflammatory responses. Regulatory macrophages, which are induced by immune complexes, produce IL-10 and IL-12 to suppress immune responses and support tumor progression. Glucocorticoid-induced macrophages secrete anti-inflammatory cytokines such as IL-10 and promote angiogenesis, which relies on anaerobic glycolysis. Tumor-associated macrophages (TAMs) facilitate tumor growth by releasing factors such as VEGF and MMP2 to promote angiogenesis and immune evasion. These diverse phenotypes highlight the dynamic and dual roles of macrophages in cancer progression and their potential as therapeutic targets. (created with BioRender)

M1 and M2 macrophages are two subtypes of macrophages that differ in their significance and roles in tumorigenesis and progression (Fig. 2). M1-type macrophages mainly perform antitumor functions, whereas M2-type macrophages mainly promote tumor cell genesis and metastasis, inhibit the immune effects of other immune cells (e.g., T cells and B cells), promote tumor angiogenesis, and assist in tissue reconstruction as well as in the repair of injuries, thereby promoting tumorigenesis and metastasis.^24^

M1-like macrophages inhibit tumor cell survival and metastasis in three main ways: first, antibody-dependent cell-mediated cytotoxicity (ADCC) action; second, antibody-dependent cellular phagocytosis (ADCP) action; and third, indirectly modulating immunity through proinflammatory factors; for example, M1-like macrophages can release proinflammatory factors such as IL-1β, IL-6, IL-12, IL-23, C-X-C motif chemokine ligand 9 (CXCL9), CXCL10, TNF-α and major histocompatibility complex (MHC) molecules. Through a constructed mouse model, Elsas et al. demonstrated that late activated M1-type macrophages are critical for effective tumor control. The expression of M1-type macrophage surface proteins (e.g., CD68 and CD80) and the intracellular protein SOCS3 are also upregulated when M1-type macrophages perform antitumor functions. Therefore, the main role of M1-type macrophages is to kill tumor cells and inhibit their growth and metastasis.^25^ In contrast, M2-type macrophages promote tumor cell proliferation and metastasis by secreting a variety of proliferation-inducing and immunosuppressive cytokines and chemokines, such as epidermal growth factor (EGF), platelet-derived growth factor (PDGF), TGF-β1, and basic fibroblast growth factor (bFGF).^24^ Moreover, M2-type macrophages also inhibit CD8+ T cells and promote the growth and proliferation of tumor cells, as well as tumor metastasis, by secreting matrix metalloproteinases (MMPs), serine proteases and histones to destroy the stromal membranes of tumor endothelial cells, as well as by secreting proangiogenic factors and enzymes involved in the regulation of angiogenesis, thus ensuring that tumor tissues have sufficient oxygen and nutrients to promote tumor growth.^26^

In recent years, M2 macrophages have been classified into four subtypes, M2a, M2b, M2c, and M2d, according to the differences in cytokines and signaling pathways involved in macrophage activation,^27–30^ and these four subtypes differ in tumor microenvironments and have their own unique functions, which will be described next. Next, we describe the differences and functions of each of these four macrophage subtypes.

In M2a macrophages, IL-4 and IL-13 are the main cytokines that can polarize macrophages to the M2a phenotype;^31^ M2b macrophages are activated primarily by the expression of high levels of anti-inflammatory cytokines in response to combined exposure to IgG immune complexes and Toll-like receptor (TLR) agonists;^32,33^ and M2c macrophages are activated by either glucocorticoid or IL-10-dependent macrophage colony-stimulating factor (M-CSF) signaling, which induces an M2 macrophage subtype.^28^ Unlike the first three subtypes, M2d macrophages are derived from polarized M1 macrophages^28^ and are induced by IL-6, TLR, and A2 adenosine receptors.^27,34^ In addition, M2b, M2c, and M2d macrophages produce more ATP through anaerobic glycolysis than do M2a macrophages, which are more dependent on the tricarboxylic acid (TCA) cycle and OXPHOS for energy supply.

Each of the four isoforms also has a unique function in the tumor microenvironment. CD206, CD209, Dectin-1, and CCL22 are known to be surface markers of M2a macrophages and are highly expressed on their surface.^28,35^ The main roles of M2a macrophages are mainly in tissue reconstruction and remodeling and in the inflammatory response,^28,32^ and their functions can be summarized in the following six aspects: 1. M2a macrophages are able to promote tissue reconstruction and remodeling through the release of a variety of cytokines and chemokines by M2a macrophages, and the released factors include IL-10, TGF-β, CCL17, CCL18, etc.^28^ In addition, some chitinase-like substances play major roles in the mechanism of reorganization;^31^ 2. M2a macrophages can promote the growth of tumor cells. M2a macrophages produce a variety of growth factors and cytokines, such as vascular endothelial growth factor (VEGF) and PDGF, which promote angiogenesis and, in turn, promote the growth of tumors.^28^ 3. M2a macrophages produce a variety of proteases, such as MMPs and histone proteases, which degrade the ECM and thus promote tumor cell invasion and migration;^28^ 4. M2a macrophages play a role in anti-inflammatory and antitumor immunity. This role is dependent on the inhibition of T-cell and NK cell activation and proliferation by anti-inflammatory factors and chemokines produced by M2a macrophages;^28^ 5. M2a macrophages release IL-4, which further promotes the polarization of more M2 macrophages toward M2a, which in turn produces more IL-4, creating a positive feedback pathway;^31^ 6. In response to IgG4, M2a macrophages can also repolarize to M2b macrophages.^27^

The function of M2b macrophages is reflected mainly in their inhibitory effect on immune responses,^28^ and they are referred to as regulatory macrophages with immunomodulatory activity.^36^ M2b macrophages play an immunomodulatory role through the release of many anti-inflammatory and proinflammatory cytokines, such as IL-6, IL-10, and IL-12,^37^ and they are able to activate Th2 cells,^27,35^ promoting naive T-cell differentiation into Treg cells. In addition, a marker of M2b cells is the high expression of CCL1, which is important for the maintenance of the M2b cell subtype.^28,31,38^ CCL1 is a chemokine that recruits NK cells, DCs, and other cells by interacting with chemokine receptor 8 (CCR8) on the cell surface^28^ and attracts CCR8-expressing Th2 and Treg cells,^35^ which promotes an immunosuppressive microenvironment that promotes tumor cell proliferation, migration, and metastasis.^35,39^ M2b macrophages have an autocrine proliferative capacity, which allows the cells to survive without the need for exogenous growth factors; this capacity is mediated by CCL1 signaling. This ability also allows M2b macrophages to survive longer in the tumor microenvironment and thus be able to sustain the suppression of antitumor immune responses.^28,38^

M2c macrophages are a subtype of anti-inflammatory M2 macrophages induced by glucocorticoid- or IL-10-dependent M-CSF signaling,^28,40^ with CD206, CD163, and Mer tyrosine kinase (MerTK) as the main markers and highly expressed on the surface of M2c macrophages.^35,41^ M2c macrophages are capable of releasing large amounts of IL-10 and TGF-β, resulting in their anti-inflammatory activity. In addition, M2c macrophages are able to produce a sustained anti-inflammatory response, which is due to the ability of M2c macrophages to produce GAS6, which is a ligand for MerTK, and when the two combine, they amplify the production of IL-10, and the large amount of IL-10 production causes M2c macrophages to exhibit anti-inflammatory activity; in addition to its anti-inflammatory activity, the release of IL-10 and the overexpression of MerTK promote its phagocytosis, removal of apoptotic cells and cell debris, etc.^28,42^ The removal of apoptotic cells not only promotes the release of anti-inflammatory factors, e.g., IL-10, from macrophages but can also trigger an anti-inflammatory response, which enhances the anti-inflammatory response; M2c macrophages also play a proangiogenic role, which is realized by M2c macrophages through the upregulation of genes, such as SPPX2 and VCAN;^27,42,43^ and M2c macrophage-released IL-10 released by M2c macrophages exerts a series of matrix remodeling, phagocytosis, and other effects by participating in signal transducer and activator of transcription 3 (STAT3)-mediated signaling, mitogen-activated protein kinase (MAPK), and other pathways.^31,43,44^ In terms of tumorigenicity, M2c macrophages play similar roles as M2a macrophages do, e.g., both exert anti-inflammatory effects, promote angiogenesis and thus tumor metastasis and invasion, promote stromal remodeling, etc.^27,31^

Unlike other subtypes, M2d-type macrophages are derived from polarized M1 macrophages, and a key factor in the polarization of M2d macrophages is an increase in extracellular adenosine levels, in addition to M2d macrophages being induced by IL-6 and TLRs.^27,29^ Both apoptotic and necrotic cells can secrete adenosine, resulting in increased extracellular adenosine levels, which induce the polarization of M2d macrophages.^28^ On the surface of M2d macrophages, phenotypes such as CD14, CD163, and CD86 are highly expressed, and CCL17 and CCL22 are expressed at lower levels.^31,45^ The protumorigenic effects of M2d macrophages are manifested in two main aspects: anti-inflammatory effects and proangiogenic effects. The anti-inflammatory effect is realized mainly through the IL-10 signaling pathway, which is activated to promote mucosal and epithelial cell healing and inflammation. In addition, IL-10 can inhibit the excessive proinflammatory response through the MAPK pathway; IL-10 also inhibits the synthesis of proinflammatory mediators, such as IL-1, IL-6, and IL-8.^27,34,46^ In terms of proangiogenic effects, M2d macrophages secrete growth factors (e.g., VEGF and PDGF) and matrix metalloproteinases (e.g., MMP2 and MMP9), which promote angiogenesis and extracellular matrix elaboration and promote tumor metastasis and growth.^28,31,45^ There is also potential communication between M2d macrophages and M1 macrophages through the VEGF and CCL3-CCR1 signaling pathways, and the polarization of M1 macrophages to M2d macrophages is facilitated through this pathway.^27,46^

In summary, the four subtypes of M2 macrophages, although differing in polarization mode and surface markers, promote tumor growth and metastasis mainly by exerting similar effects, such as anti-inflammatory and angiogenesis-promoting effects.

Chen et al. investigated the effect of M2 macrophages on cancer cell metastasis. They cocultured M1 and M2 macrophages with gastric cancer cells, breast cancer cells and melanoma cells and reported that the number of migrated cancer cells cocultured with M2 macrophages increased significantly and that coculture of M1 macrophages with cancer cells did not affect the number of migrated cancer cells, suggesting that M2 macrophages play an important role in the migration of gastric cancer and breast cancer cells. Migration of gastric cancer and breast cancer. In addition, this research team experimentally reported that the CHI3L1 protein secreted by M2 macrophages may play an important role in promoting cancer cell metastasis.^47^

Macrophages can differentiate into two types, M1 and M2, due to the influence of different cytokines and metabolites in the TME. This classification was initially proposed on the basis of the stimulatory response of macrophages to type 1 or type 2 cytokines in in vitro experiments,^48,49^ but with the continuous development of technology and the deepening understanding of macrophages, we have shown that macrophages have a high degree of plasticity and heterogeneity and that they can reregulate their phenotype in response to different bits of environmental stimuli. Therefore, it is overly simplistic to categorize them as M1 and M2 only.^50,51^

Single-cell sequencing is a technology that sequences and analyzes genomes, transcriptomes and epigenomes at the single-cell level. Single-cell sequencing technology has been widely utilized because it is a good solution to the problem of loss of information on cellular heterogeneity caused by the traditional technique of sequencing on a multicellular basis. In recent years, many researchers have discovered new tumor-associated macrophage subtypes via single-cell sequencing technology, and these subtypes play important roles in tumor growth and proliferation, which affects the prognosis of patients. Q. Zhang, Y. He et al. collected six macrophage clusters in hepatocellular carcinoma and reported that among them, THBS1+ macrophages (myeloid-derived suppressor cell (MDSC)-like macrophages) and C1QA+ macrophages (TAM-like macrophages) were enriched in tumor tissues; these features are similar to those of TAMs but more complex than those of the classical M1/M2 model is, thus identifying these two types of macrophages as new subtypes.^52^ Zhang et al. also used single-cell sequencing to identify a new macrophage subtype, FABP4+C1q+, in which two genes, fatty acid binding protein 4 (FABP4) and the complement C1qA chain, are highly expressed and serve as marker genes. In addition, the team reported that FABP4+C1q+ macrophages focus on positive regulation of cytokine production, the inflammatory response, chemokine production, neutrophil activation, leukocyte chemotaxis and migration and have increased proinflammatory cytokine secretion, phagocytosis, and antiapoptotic functions. These effects may constitute one of the main mechanisms by which FABP4+C1q+ macrophages exert antitumor effects. The antitumor capacity of FABP4+C1q+ macrophages was also verified by a team that used tumor tissues from non-small cell lung cancer (NSCLC) patients and reported that there was a better prognosis with FABP4+C1q+ macrophage enrichment.^53^ In glioma cells, Xu et al. identified a macrophage subtype with high FN1 gene expression, defined it as FN1+ TAMs, and reported that FN1+ TAMs play a key role in glioma recurrence.^48^ In addition, in melanoma, Wu et al. identified and obtained a subpopulation of MerTK+ macrophages and reported that this subpopulation has potent immunosuppressive activity that promotes tumor growth.^54^

Using single-cell sequencing technology, multiple novel macrophage subpopulations can be identified, and they influence tumor progression through different signaling pathways; for example, aryl hydrocarbon receptor (AHR)-ALKAL1 signaling is a key regulator of the MerTK+ macrophage subpopulation,^54^ and in-depth studies of these novel macrophages may be able to identify new targets for tumor therapy.

The difference in the surface markers of TAMs is also one of the reasons why M1 and M2 macrophages play different roles in tumor tissues. Notably, although there are some common TAM surface markers, such as CD11b, CD11c, and CD64, in humans and mice, there are still differences. In humans, the TAM marker is the universal marker CD68, whereas in mice, it is the specific universal marker F4/80.^55^ Qiao T et al. reported that F4/80 TAMs are close to neovascularization and tumor vessels and are prone to angiogenesis in vivo. It also strongly promoted the activation of vascular endothelial growth factor A (VEGFA), Ki67 and other key angiogenesis markers in endothelial cells in vitro.^56^ H.H. Lin et al. reported that the F4/80 molecule plays a crucial role in the development of Ag-specific regulatory T cells that can inhibit Ag-specific immunity, providing direct evidence for its role in the induction of Ag-specific tolerance.^57^ By searching the relevant data, we summarized and drawn a table of the main types of surface markers of M1 and M2 macrophages, the different roles of different surface markers and the related pathways (Table 1) so that we can further explore the mechanisms underlying the different meanings.

**Table 1:** Surface markers and functions of M1 and M2 macrophages

TAM marker	Cell type	Overall functionality	Change in TAMs	Therapeutic drugs
CD80	M1	Anti-tumor	Promote T-cell activation and proliferation; increase T-cell infiltration; block immunosuppression of tumor cells by binding to PD-1, etc.	
CD86	M1	Anti-tumor	Promote T-cell activation and proliferation; increase T-cell infiltration; enhance the effects of immune checkpoint inhibitors, etc.	
CD40	M1	Anti-tumor	Increase in macrophage infiltration of tumors; promotion of the tumor-killing effect of macrophages; promotion of tumor stroma reduction, etc.	
MHC- II	M1	Anti-tumor	Activation of naive CD4+ T cells; increased infiltration of lymphocytes into tumors; improved overall survival of tumor patients, etc.	
TLR2	M1	Anti-tumor and pro-tumor	TLR2plays different roles in the presence or absence of binding adjuvant and in the presence of different adjuvants	minocycline
TLR4	M1	antitumor	Through interaction with CCRL2, the activation of macrophages and the promotion of subsequent anti-tumor responses by CD8+ T cells, among other things	
CD274	M2	tumor promoting	Binds to PD-1 and inhibits T-cell-mediated anti-tumor immune responses in the tumor microenvironment, leading to poor prognosis, among others	Aspirin
CD206	M2	Tumor-promoting as the main focus	When CD206+TAM has antigenic cross-competence, it improves overall survival in tumor patients; high CD206macrophage infiltration is associated with advanced stage and high tumor recurrence rates, among others	1 Pulsatilla chinensis (P.chinensis) 2. Ginsenoside Rh2(G-Rh2)
CD163	M2	tumor promoting	The CD163 macrophage population has anti-inflammatory functions and promotes angiogenesis, making malignant tumors more susceptible to further metastasis, leading to a poorer prognosis and reduced overall survival	1. diHEP-DPA 2. Epimedium Extract 3. Azithromycin polymeric prodrug nanoparticles modified by CDl63 monoclonal antibody
CD204	M2	tumor promoting	CD204is involved in tumor phagocytosis processes and reactive oxygen species production, and CD204-positive M2 macrophages increase cancer cell migration	1. Vitamin D3 2. Leiogangenoside
FOLR2	M2	antitumor	FOLR2 macrophages in tumors are often tissue-resident macrophages; FOLR2 macrophages are able to interact with CD8+ T cells to provide energy to CD8+ T cells and accelerate the induction of apoptosis in tumor cells, positively associated with T-cell infiltration and better prognosis	
CD44	M1/M2	tumor promoting	CD44 is associated with lymph node metastasis and protein digestion and uptake, and promotes the proliferation and migration of tumor cells.	1. Combination therapy with celecoxib and epirubicin 2. Hyaluronic acid-modified metastases loaded with chlorogenic acid (CHA)

### Distribution of TAMs in the tumor immune microenvironment

TAMs are the most abundant cell population in the tumor immune microenvironment, and TAM infiltration is closely associated with tumor stage and metastasis. Zheng et al. studied 104 lung cancer patients and reported that the density of M1-type TAMs was greater than that of M2-type TAMs and that the density of M2 invasive margin (IM)-TAMs was significantly greater.^58^ There was no significant difference in the density of M1 TAMs between the tumor center (TC) and IM regions. TAMs infiltrated more of the stroma than the parenchyma in both the M1 and M2 types. Patients with high-density M1 TAMs had greater overall survival (OS) benefits, whereas M2 TAM density was not significantly associated with overall survival. The probability of metastasis significantly increased with increasing proximity of the tumor to M2 TC-TAMs or M2 IM-TAMs, and tumor size was correlated with the proximity of M2 IM-TAMs, with larger tumors being closer to each other.

Every adult tissue contains a rich population of resident macrophages; for example, in the breast, there are at least two resident macrophage populations: stromal and ductal macrophages (SM and DM, respectively). Laviron et al.^59^ identified several TAM subpopulations in breast tumors that exhibit different ecological niches from those of macrophage subpopulations prior to tumor development and reported that the TAM composition shifts between proliferative and malignant neoplastic lesions; for example, the expression of CD11b is significantly elevated in malignant neoplastic lesion-associated enhanced green fluorescent protein (EGFP) cells compared with that in proliferative lesions, which is consistent with the CD11b phenotype of macrophages in the pretumor tissue epithelium. The classification of M1, M2-type macrophages has already been widely used for the classification of TAMs; however, a growing body of research and evidence now suggests that this classification is oversimplified and that macrophages exhibit different transcriptomes in response to different external stimuli, and TAM heterogeneity has been described in different tumor models. Huang et al. investigated the relationship between the tumor environment and the heterogeneity of TAMs via multiplex immunohistochemistry in 56 gastric cancer (GC) cases.^60^ There was an increase in M2-type TAMs at the edge of the tumor and an increase in M1-type TAMs in the core. The study classified TAMs into seven populations and revealed that CD68+, CD68+CD206++ and CD68+CD163+CD206+ cells were enriched in all regions of interest. CD68+CD163+CD206+ cells accumulated the most at the margins, decreasing toward the core, whereas the opposite was true for CD68+IRF8+ cells. CD68+CD163+ and CD68+CD206+ cells were more densely packed in tumors than in normal tissue. These findings suggest that TAMs polarize according to their location. In addition, the macrophage count was correlated with patient recurrence-free survival (RFS) and OS. These findings indicate that the distribution of TAMs is different in different tumor immune microenvironments and that the different distributions of TAMs affect tumorigenesis and progression, which in turn affects the prognosis of patients. We may be able to treat tumors by regulating the spatial distribution and number of TAMs in the TME.

### Heterogeneity of TAMs exerting antitumor and protumor effects

Macrophages are highly heterogeneous, functionally plastic immune cells, and their complexity and heterogeneity increase accordingly with tumor progression.^61,62^ Depending on the microenvironment in which they reside, they can play either an immune-supportive or a tumor-supportive role.^63,64^ This antitumor and protumor heterogeneity of TAMs is reflected mainly in the fact that macrophages can be polarized into two phenotypes depending on the environment in which they live: M1-type macrophages and M2-type macrophages. These two subtypes represent the two extremes of the macrophage functional spectrum,^65^ and the differences in the role and distribution of these two cell types were mentioned earlier. Since macrophages can change their M1 and M2 status according to different stimuli in the different environments in which they find themselves,^66^ studies have been conducted to convert macrophages from the protumorigenic M2 type to the antitumorigenic M1 type via pharmacological or other methods, thereby improving the immunotherapeutic effect on tumors.^67^

## TAM signaling pathways and multilayered regulatory mechanisms

**Fig. 3:**
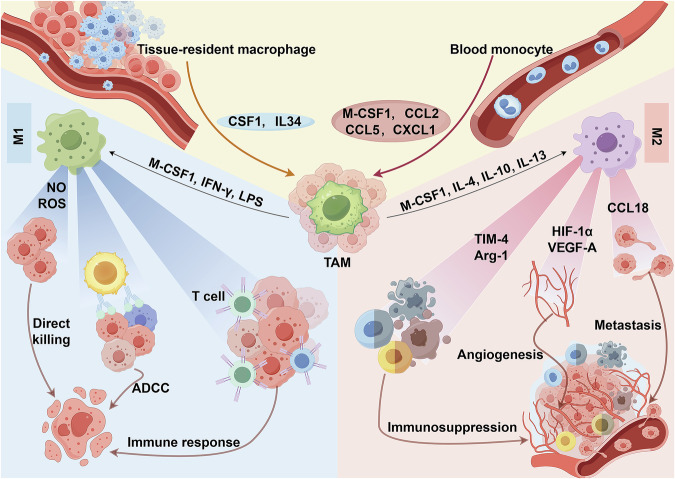
Distribution and density of tumor-associated macrophages in the tumor microenvironment. The spatial distribution of TAMs in tumor tissues, with a focus on the contrasting densities of M1 and M2 TAMs in various tumor regions. The figure shows that M1 TAMs are generally found at higher densities in central tumor regions, promoting antitumor immunity, whereas M2 TAMs are more prevalent in the periphery, assisting in tumor growth and metastasis. (created with Figdraw)

TAMs have dual influences as both promoters of tumorigenesis and designers of immunosuppressive tumor microenvironments, which allows them to fight against tumor cells and, at the same time, may promote tumor growth and spread (Fig. 3). This dual role depends mainly on the two subtypes of TAMs: type M1 and type M2. Type M1 TAMs are usually associated with an antitumor immune response, with the ability to promote inflammation and kill tumor cells. In contrast, M2-type TAMs are usually associated with the suppression of immune responses, the promotion of tumor growth, and the support of angiogenesis.

### TAM signaling pathways

Tumor-associated macrophages are important immune cells in the tumor microenvironment, and they play a key role in tumorigenesis, progression, and metastasis. TAMs interact with tumor cells through a variety of signaling pathways to promote tumor progression. The following are some of the key signaling pathways associated with the function of TAMs.

#### Proinflammatory signaling pathway

The macrophage phenotype is plastic and can change in response to cytokines, cell‒cell interactions and tissue-specific signals. Immunosuppressive molecules and inhibitory pathways, including mechanistic targets of the NF-κB and rapamycin (mTOR) signaling pathways, are involved in macrophage differentiation. TAMs respond to sufficient upstream activation signals to produce abundant reactive oxygen species (ROS), which subsequently mediate immunosuppressive activity via the NF-κB pathway. It is hypothesized that the NF-κB pathway manipulates signal activation in cancer cells and tumor-infiltrating leukocytes to promote inflammatory responses in the TME. For example, Clec4e molecules can activate the NF-κB signaling pathway via Syk kinase, which in turn promotes the protumorigenic effects of TAMs.^68^ Therefore, blocking TNF-α with anti-TNF-α antibodies may be therapeutically useful.^69^ IFN-γ is an anti-inflammatory factor that inhibits the production of TAMs, thereby reversing the immunosuppressive and tumorigenic properties of TAMs,^70^ whereas the NF-κB signaling pathway is involved in the regulation of macrophage activation, which mediates cytotoxicity against tumor cells.^71^

Another pathway that promotes inflammation and tumor cell proliferation is the STAT pathway. TAMs have multiple effects on the STAT signaling pathway, and they can activate or inhibit the STAT signaling pathway by secreting cytokines and growth factors, thereby affecting tumor growth and progression. TAMs can secrete a variety of cytokines, such as IL-6 and IL-10, which can activate Janus kinase (JAK), which phosphorylates STAT3, causing it to form a dimer and translocate to the nucleus, initiating the transcription of downstream genes. The activation of STAT3 promotes the expression of a variety of genes, including VEGF, MMPs, and cyclin D1, which are associated with tumor angiogenesis, invasion, and progression. tumor angiogenesis, invasion and proliferation. Studies have shown that STAT3 can also promote the expression of programmed death-ligand 1 (PD-L1), which inhibits the antitumor activity of T cells. One study revealed that in ovarian cancer, STAT3-activated TAMs can express PD-L1 and bind to programmed cell death protein 1 (PD-1) receptors on T cells, leading to T-cell inactivation.^72^ The activation of STAT5 can promote the expression of various genes, including Bcl-xL and Mcl-1, which are related to cell survival and proliferation.

#### Proangiogenic signaling pathway

Angiogenesis is an important process in tumor biology and plays a key role in promoting tumor nutrition and oxygen supply; metabolism and growth; invasion and metastasis; and remodeling of the tumor microenvironment. Among them, the VEGF pathway and hypoxia-inducible factor 1α (HIF-1α) pathway are strongly influenced by TAMs, which can secrete VEGF, activate the VEGF pathway, and promote tumor angiogenesis. Moreover, HIF-1α is upregulated in TAMs, which can increase VEGF expression and further promote angiogenesis. Therefore, inhibiting the angiogenic process has become an important strategy in tumor therapy, by which angiogenesis can be inhibited to slow or stop tumor growth and metastasis. For example, antiangiogenic drugs such as Avastin and Sorafenib have been used to treat certain types of cancer.

#### Extracellular matrix remodeling signaling pathway

MMPs are a group of protein hydrolases capable of degrading the ECM; they play a key role in tumor invasion, metastasis, and angiogenesis, and the action of TAMs on the MMP pathway involves a variety of mechanisms. TAMs can activate the production and secretion of MMPs by secreting a variety of cytokines, such as TNF-α, IL-1β, and TGF-β, to activate the production and secretion of MMPs. One study revealed that TAMs can induce the production of MMP-2 and MMP-9 via IL-1β.^73^ Moreover, certain proteases secreted by TAMs, such as histone B (Cathepsin B), can activate inactive pre-MMPs (e.g., pre-MMP-2 and pre-MMP-9) to become active forms. Active MMPs can degrade the ECM, providing a pathway for tumor cell invasion and metastasis. TAM-mediated activation of the MMP pathway also promotes the invasion and metastasis of breast cancer tumor cells.

#### Immunosuppressive signaling pathways

First, TAMs can express PD-L1, one of the ligands for PD-1. PD-L1 binds to the PD-1 receptor on T cells and inhibits T-cell activation and function. One study revealed that in ovarian cancer, TAMs express PD-L1 and bind to the PD-1 receptor on T cells, leading to T-cell inactivation.^74^ TAMs also express the PD-1 receptor. The activation of PD-1 can inhibit the phagocytosis and clearance of TAMs, which can affect the immunosurveillance role of TAMs. A previous study revealed that the inhibition of PD-1 enhances the phagocytosis of TAMs, thereby prolonging survival time in a mouse model of colon cancer.^75^ In addition, TAMs can interact with T cells and inhibit the antitumor immune response of T cells through the PD-1/PD-L1 signaling pathway. This interaction can lead to T-cell depletion and dysfunction, thereby promoting tumor growth and metastasis.

Second, the action of TAMs on the cytotoxic T-lymphocyte-associated protein 4 (CTLA-4) pathway involves multiple mechanisms. CTLA-4 is an immune checkpoint molecule that is expressed on tumor cells and immune cells and inhibits the activation and proliferation of T cells. TAMs can express CTLA-4 and bind to the CTLA-4 ligands B7.1 and B7.2 on T cells, which inhibits T-cell activation and proliferation.^76^ This inhibition reduces the killing of tumor cells by T cells, thereby promoting tumor growth. TAMs can also secrete immunosuppressive molecules, such as IL-10 and TGF-β, which can act synergistically with the CTLA-4 pathway to further enhance the immunosuppressive effect.

#### Other pathways

In terms of cell survival and proliferation, TAMs promote cell survival and proliferation by activating the PI3K/Akt pathway while inhibiting apoptosis.^77^ TAMs also activate the Ras/ERK pathway, which is involved in cell proliferation and differentiation.^78^ In terms of the cell‒cell interaction signaling pathway, TAMs interact with the extracellular matrix via integrins to influence cell adhesion and migration. In addition, TAMs have intrinsic signaling pathways, i.e., the CSF-1/CSF-1R pathway. CSF-1 binds to its receptor (CSF-1R) and is a key regulator of TAM survival and function.^79^

The activation and regulation of these signaling pathways in TAMs are very complex, and they are intertwined with each other, not only affecting tumor growth and metastasis but also participating in the development of the tumor microenvironment, which plays an important role in immune escape and therapeutic resistance. Therefore, an in-depth understanding of these signaling pathways can help in the development of new tumor therapeutic strategies to improve the efficacy of treatment by regulating the function of TAMs.

### Role of TAMs in tumorigenesis

#### Tumor promotion

During tumor progression, acute and chronic inflammation, wound healing, and the female reproductive cycle, the original vascular system teeth out epithelial cells to form new blood vessels via a process called angiogenesis. During angiogenesis, factors such as VEGFs and placental growth factor (PIGF) stimulate quiescent endothelial cells to release proteases such as MMP-9, thereby reducing intercellular adhesion and degrading the basement membrane. Disruption of intercellular adhesion and the continuous basement membrane results in blood vessels that are not in their normal state, become distorted or inflated, and are more prone to invade the tumor microenvironment and become locally hypoxic. Tumors require a vascular bed formed by endothelial cells to provide nutrients and oxygen and carry away waste and carbon dioxide.^80^

Tumor-associated macrophages are involved mainly in tumor angiogenesis through the following three mechanisms: (1) the hypoxic tumor microenvironment stimulates macrophages to overexpress HIF, which acts as a transcription initiation factor that binds to the promoters of target genes, such as VEGF-A, and induces the expression of VEGF-A;^81^ macrophages express factors such as IL-1β, TGF-β, and TNF-α, which stimulate fibroblasts and adenocarcinoma cells to express VEGF;^82^ (2) macrophages are capable of secreting proteases such as MMP-7,^83^ MMP-9,^84^ and MMP-12;^85^ and (3) macrophages may also differentiate into endothelial-like cells (expressing Tie2), which reside at sites of intense angiogenesis and promote angiogenesis through the expression of VEGF-A, MMP-9, and cyclooxygenase-2 (COX2).^86,87^

In addition, TAMs can help tumors evade immune surveillance by suppressing adaptive immune responses. Organisms can inhibit tumorigenesis and development through natural and acquired immunity, while tumor cells can evade recognition and attack by the organism’s immune system through a variety of mechanisms. The inhibition of antitumor immunity by TAMs involves the following mechanisms: (1) tumor cells produce IL-10, which induces the expression of PD-L1 on the surface of TAMs,^88^ binds to PD-1 on the surface of T cells in the TME, and inhibits cytotoxic T-cell function; (2) TAMs produce CCL22, which recruits regulatory T cells into the TME and inhibits the activation and function of effector T cells;^89^ and (3) TAMs produce Arg-1, which catalyzes the hydrolysis of L-arginine to urea and L-ornithine, inhibits the upregulation of cytokinin D3 and cytokinin-dependent kinase 4, and prevents the T-cell cycle from proliferating by arresting in the G0/G1 phase.^90^ Overall, TAMs play dual roles as “immune suppressors” and “tumor promoters” because they can promote tumorigenesis and act as central drivers of the immunosuppressive TME.

#### Tumor suppression

The process of specific recognition and clearance of tumor cells by immune cells is complex, and macrophages are among the most important members of this process. TAMs are key components of leukocyte infiltration and are widely observed in a variety of tumors. In most studies, the density of TAMs has been found to be associated with poor prognosis in cancer patients,^91^ whereas very few studies have shown that the density of TAMs in the TME is associated with good prognosis.^92^ This duality has been found in a variety of cancers, including prostate, lung, and brain cancers.^93^ Other researchers have reported that TAMs inhibit the growth and metastasis of osteosarcoma and are associated with a favorable prognosis. Both the M1 and M2 isoforms of TAM inhibit the growth of osteosarcoma cells under certain conditions.^94^ The dual nature of the TME may be due to the influence of other cell types present within the TME.

First, macrophage-mediated programmed cell removal (PrCR) plays an important role in tumor elimination and monitoring. The activation of the TLR pathway in macrophages induces the activation of Bruton’s tyrosine kinase (Btk) signaling pathway,^95^ which dissociates from endoplasmic reticulum cell surface calreticulin (CRT) phosphorylation. The dissociated CRT is expressed on the surface of macrophages, which then forms the CRT/CD91/C1q compound, which targets cancer cells for phagocytosis.^96^ Second, activated macrophages can also defend against tumors by directing tumor cytotoxicity and secreting cytokines. For example, M-CSF and muramyl dipeptide (MDP) are added to macrophages in in vitro culture to enhance macrophage cytotoxicity, or immunomodulators are loaded by intravenous injection of liposomes to increase macrophage toxicity. Molecules of microbial agents and pathogens can also stimulate antitumor cytotoxicity in macrophages, as in the case of the use of Bacillus Calmette-Guerin (BCG) in the treatment of bladder cancer, which increases macrophage cytotoxicity against certain bladder cancer cell lines by stimulating macrophages.^97^ Moreover, many studies have shown that TAMs have the ability to phagocytose and remove damaged cells. In the early stage of tumorigenesis, TAMs can phagocytose and remove abnormal or damaged cells, preventing them from developing into tumor cells.^98^ Moreover, TAMs can degrade the extracellular matrix by secreting MMPs, a process that is usually promoted in tumorigenesis. However, studies have demonstrated that under appropriate conditions, MMPs can promote the clearance of tumor cells. Moreover, TAMs may inhibit the self-renewal ability of tumor stem cells by secreting antitumor factors, regulating metabolic pathways, suppressing stemness gene expression, and modulating immunosuppressive cells, thereby reducing tumorigenesis.^99^

### Regulation of TAMs in the metastasis process

Wang et al.^100^ reported that coculturing macrophages and several non-small cell lung cancer cell lines in vitro increased the matrix degradation activity and invasion ability of these lung cancer cells, suggesting an important role for TAMs in the invasion and metastasis of non-small cell lung cancer. Cell migration, which generally refers to the movement of individual cells, consists of 4 steps: the cell front extends a lamellar pseudopod; the cell front pseudopod and extracellular matrix form a new cell adhesion; the cell body shrinks; and the cell tail and surrounding matrix adhesion dissociates and the cell moves forward. Cancer cells generally migrate in groups, called “collective cell migration”, during which tumor cells form cell scaffolds at the front of the migration site through cell adhesion molecules, such as integrins and calcineurin, to pull other cells forward, a process that requires the protein hydrolases MMP-14, MMP-2, and MMP-9 to play a role.^101,102^

TAMs promote tumor migration and infiltration mainly through these mechanisms. First, as mentioned earlier, TAMs promote tumor angiogenesis, which involves the secretion of a variety of proangiogenic factors, such as VEGF, bFGF, and PDGF, which promote tumor vascularization and provide the necessary nutrients and oxygen for tumor growth and metastasis. Second, TAMs can remodel the extracellular matrix by secreting a variety of MMPs, such as MMP-2, MMP-9, MMP-3, and MMP-7,^103^ which are enzymes that degrade the extracellular matrix and make it easier for tumor cells to invade and metastasize, and TAMs can also secrete TNF-α and TGF-β, which induces EMT, which endows tumor cells with more loose cellular connectivity and accelerates the movement of tumor cells.^104^ TAMs inhibit antitumor immune responses by secreting immunosuppressive factors such as IL-10, TGF-β, and prostaglandin E2 (PGE2), which contributes to the immune escape and metastasis of tumors. Moreover, M2-type macrophages have significant immunosuppressive effects and have been found to secrete immunosuppressive molecules, including IL-10, TGF-β and human leukocyte antigen G (HLA-G), into the TME.^105^ In addition, M2-type cells interact directly with MDSCs and actively inhibit T-cell-mediated antitumor responses.^106^ Immunosuppressive cells, such as Treg cells, indirectly inhibit T-cell activity. Furthermore, TAMs can directly inhibit the proliferation of CD8+ T cells by metabolizing L-arginine via arginase-1, inducible nitric oxide synthase (iNOS), oxygen-free radicals, or nitrogen species.^107^

More importantly, when macrophages express CSF-1R, tumor cells secrete M-CSF to attract TAMs,^108^ and after TAMs are attracted to tumor cells, they secrete EGF, which activates the epidermal growth factor receptor (EGFR) signaling pathway in tumor cells, and the activation of the EGFR pathway results in the extension of more pseudopods in tumor cells.^101^ Since myeloid cells are highly mobile and less compact, tumor cells combined with TAMs can gain stronger metastatic ability and are more likely to metastasize to the distal end.

Recently, researchers have reported that TAMs, which are characterized by an M2-polarized phenotype, can promote the metastasis of gastric cancer cells through exosomes.^109^ TAMs can deliver exosomes to tumor cells, which are enriched in miRNAs, lncRNAs, and specific proteins that promote tumor metastasis.^110^ Therefore, in malignant tumors, exosomes serve as important carriers for the exchange of substances and information in the tumor microenvironment, are involved in different stages of cancer cell survival and growth as well as tumor metastasis, and can be used as targets for tumor immunotherapy.^111^

In summary, TAMs play multiple facilitating roles in the process of tumor metastasis, helping tumor cells evade immune surveillance, invade surrounding tissues, enter the blood circulation and colonize distant organs to form metastatic foci through multiple mechanisms.

### Regulation of TAMs by organelle signaling

Cell signaling plays a crucial role in regulating macrophage function, particularly in tumor immunity and disease progression. Studies have shown that pyroptosis induced by photocatalytic carbon dots can significantly increase the antigen-presenting capacity of macrophages, thereby triggering specific tumor immune responses and providing new insights for tumor immunotherapy.^112^ Furthermore, clusterin (CLU) promotes the polarization of macrophages toward the M1 phenotype by inducing mitochondrial damage and activating the type I interferon pathway, thus enhancing their antitumor capabilities.^113^ This mechanism further highlights the central role of organelle signaling in tumor immune responses.

With respect to the metabolic regulation of macrophages, M1 macrophages exhibit downregulation of c-Myc expression under proinflammatory stimuli, which inhibits proliferation while upregulating HIF-1α and glycolysis. In contrast, M2 macrophages upregulate c-Myc, promoting their differentiation toward the anti-inflammatory phenotype.^114^ These metabolic changes determine the immune function of macrophages in the tumor microenvironment, thereby influencing the aggressiveness and metastatic potential of tumors.

In addition, the mechanism by which LC3-associated phagocytosis (LAP) regulates macrophage phagocytic function in acute myeloid leukemia (AML) has been elucidated. Loss of LAP leads to increased tumor burden and shortened survival, whereas activation of the stimulator of interferon genes (STING) signaling pathway inhibits tumor growth by increasing the phagocytic potential of macrophages. In AML, the antitumor effect of STING differs from its role in solid tumors; STING primarily exerts its antitumor effect by enhancing the phagocytic ability of macrophages.^115^

Moreover, Caspase-1 enhances the protumor function of TAMs by specifically cleaving Peroxisome proliferator-activated receptor gamma (PPAR-γ), while tumor cells counteract phagocytosis by overexpressing glutamine-fructose-6-phosphate transaminase 2 (GFPT2), thereby inhibiting macrophage mitochondrial fission.^116^ These studies not only underscore the importance of organelle function and signaling pathways in the interaction between macrophages and tumor cells but also reveal the potential of these pathways as therapeutic targets for cancer treatment.

### Role of TAMs in tumor recurrence and resistance to therapy

Drug resistance is a challenge for many tumor chemotherapy regimens. In pancreatic ductal adenocarcinoma (PDA), TAMs can release deoxycytidine, which inhibits gemcitabine at the level of drug uptake and metabolism through molecular competition, leading to resistance to gemcitabine in PDA.^117^ Similarly, TAMs can secrete large amounts of IL-1β under stimulation with cisplatin (CDDP), a neoadjuvant chemotherapeutic agent for osteosarcoma, which reduces osteosarcoma cell sensitivity to CDDP and leads to drug resistance.^118^ Therefore, TAM-mediated tumor cell resistance may be an important reason for the stagnation of neoadjuvant chemotherapy.^119^

CSCs, also known as tumor-initiating cells or tumor-maintaining cells, constitute a stem cell-like subpopulation within the tumor cell population.^120^ CSCs are highly resistant to chemotherapy and radiotherapy. The removal of CSCs reduces tumor resistance and thus prevents tumor recurrence.^121^ TAMs can directly interact with CSCs and maintain the stem cell-like characteristics of CSCs, thereby triggering tumorigenesis and tumor progression.^122^ In addition, CD209-positive M2-type TAMs were found to activate CSCs and promote osteosarcoma formation, whereas all-trans retinoic acid (ATRA) inhibited in vitro osteosarcoma cell colony formation and spheroidogenic capacity as well as TAM-induced osteosarcoma formation in mice in vivo by decreasing the activity of CSCs and inhibiting M2-type TAMs.^123^

There is also substantial evidence that TAMs promote tumor growth by promoting angiogenesis, immunosuppression, and chronic inflammation and can influence tumor resistance after conventional anticancer therapy, thus further promoting tumor recurrence. TAMs can inhibit antitumor immune responses through the secretion of immune-suppressive factors such as IL-10 and TGF-β, thus providing an opportunity for tumor cells to survive and recur after treatment. In a study on melanoma, researchers reported that the number of TAMs was associated with an increased rate of tumor recurrence. TAMs reduce the likelihood of tumor clearance by inhibiting the activity of CD8+ T cells.^124^

In addition, TAMs can increase the resistance of tumor cells to chemotherapy and targeted therapy by secreting antiapoptotic factors, regulating the cell cycle, promoting DNA repair, etc. IL-10 secreted by TAMs can inhibit Fas/FasL-mediated apoptosis, thus protecting tumor cells from being killed by chemotherapeutic drugs. Moreover, TGF-β secreted by TAMs can inhibit the expression of the cell cycle protein-dependent kinase (CDK) inhibitor p27, leading to an uncontrolled cell cycle and increased drug resistance in tumor cells. A study on lung cancer revealed that TAMs promote the migration and invasion of tumor cells through the secretion of cytokines such as IL-6 and IL-8 and simultaneously increase the resistance of tumor cells to EGFR inhibitors,^18^ reducing the efficacy of chemotherapeutic drugs. Therefore, therapeutic strategies targeting TAMs, such as inhibiting their immunosuppressive activity or promoting their antitumor function, may help reduce tumor recurrence and improve the effectiveness of cancer therapy.

## TAMs interact with immune cells

**Fig. 4:**
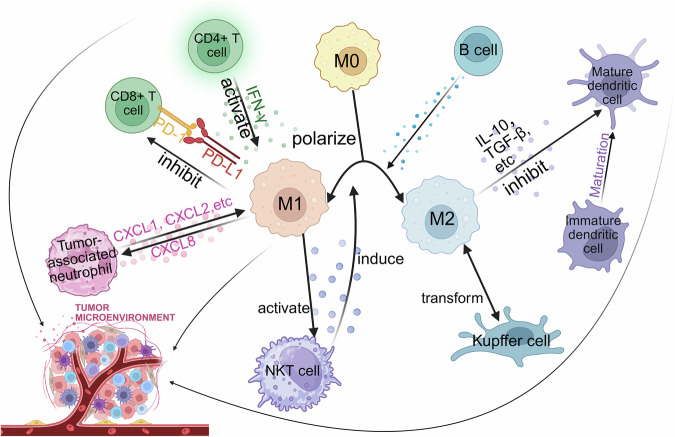
Immune interactions of tumor-associated macrophages in the tumor microenvironment. Schematic representation of the interactions between tumor-associated macrophages (TAMs) and various immune cells within the tumor microenvironment (TME). TAMs polarize into M1 (proinflammatory, antitumor) or M2 (anti-inflammatory, protumor) phenotypes, which are influenced by cytokines such as IFN-γ and IL-10. They interact with CD4+ T cells, CD8+ T cells, and regulatory T (Treg) cells through cytokines and immune checkpoints such as PD-L1, modulating immune responses. TAMs also affect dendritic cells (DCs), neutrophils (TANs), B cells, and Kupffer cells by altering their activation, recruitment, and polarization states. These interactions collectively contribute to the suppression of antitumor immunity and the promotion of tumor growth and metastasis. (created with BioRender)

As major components of TME, TAMs interact with immune cells including T cells, dendritic cells, Tumor-associated neutrophils, B cells, Kupffer cells. The interaction between TAMs and other immune cells is briefly illustrated in Fig. 4.

### T cells

#### CD4+ T cells

In the early stages of tumor development, since free radicals produced by macrophages often lead to DNA damage, which in turn causes mutations and host cell transformation, it is widely believed that in the early stages of cancer development, macrophages exist in a form similar to classically activated macrophages with an inflammatory phenotype, contributing to the early eradication of transformed cells.^125^ However, as the tumor progresses and grows, the tumor microenvironment significantly influences the onset/development of TAMs, and these macrophages take on a phenotype that more closely resembles that of regulatory macrophages.^126^ Regardless of the stimulus, this new class of macrophages produces high levels of IL-10 to inhibit immune responses to neoantigens expressed by tumor cells and can inactivate neighboring macrophages.^127,128^ It has also been demonstrated that regulatory macrophages promote angiogenesis, which in turn promotes tumor growth.^129^

Some studies have classified mouse Th lymphocytes into Th1 and Th2 cells on the basis of their respective cytokine production (IFN-γ and IL-4).^130^ These cytokines have cross-regulatory properties that are critical for triggering IFN-γ production and Th1 cell development or IL4/IL-13 secretion and Th2 cell proliferation.^81,131^ IL-12 or IL-10 production likewise sets the stage for M1/M2-type cell polarization.^62,132–134^

IFN-γ is the only type II IFN that is recognized by a receptor consisting of two ligand-binding IFN-γ receptor 1 (IFNGR1) chains and two signaling IFNGR2 chains.^135^ It is now well established that IFN-γ is involved in macrophage “initiation” by increasing macrophage responses to inflammatory molecules such as Toll-like receptor ligands and tumor necrosis factor.^136^ Muller et al. demonstrated that IFN-γ synergized with Toll-like receptor ligands to induce tumor-killing activity in preconditioned macrophages and enhanced the expression of TNF-α and IL-12 family cytokines.^137^ Furthermore, in the TME, IFN-γ produced by cytotoxic immune cells increases the number of iNOS(+)CD206(-) M1-type macrophages, thereby inhibiting tumor growth.^138^ iNOS(+)CD206(-) M1-type macrophages have been shown to be correlated with a favorable prognosis in a variety of tumors, such as breast, lung, ovarian, and gastric cancers.^139–142^ On the other hand, M1-type TAMs in the TME secrete CXCL9, CXCL10, and CD86, which stimulate CTL recruitment to the TME and its activation, and recruit CTLs to produce IFN-γ, which is equally important for maintaining M1-type TAM activity and tumor suppression.^143^ However, IFN-γ also induces the apoptosis of CD4+ T cells and reduces secondary antitumor immune responses.^144^

While the immunity of Th2 cells to parasites and the pathogenic role of allergic diseases are well established, the regulation and function of Th2 cells in the TME remain largely neglected and controversial. In general, Th2 cell immunity to tumors is mediated by Th2 cytokines and acts synergistically with macrophages through secondary recruitment of tumor-killing myeloid cells, such as eosinophils.^145,146^ It has been demonstrated that mice deficient in the Th2 cytokines IL-2 and IL-4 show reduced tumor clearance.^147^ Neutralization of IL-4 by monoclonal antibodies may also help restore tumor growth.^148–152^ The possible mechanism involves the secretion of the cytokines IL-4 and IL-13 by Th2 cells, which prompts the transformation of TAMs into M2-type macrophages, which in turn promotes tumor growth.

Th2 cytokine production and type 2 immunity are also mediated by type 2 intrinsic lymphocytes (ILC2s). ILC2s also secrete Th2 cytokine and are dependent on the Th2 cell transcription factor GATA binding protein 3 (GATA3) but lack TCR expression.^153–155^ Notably, Th2 cells in the TME correlate with the progression of breast and cervical cancers.^156–158^ In addition, type 2 immunity, largely driven by Th2 cells, has been shown to promote tumor metastasis in breast, colorectal, and lung cancers.^159–162^

Th17 cell development is distinct from that of Th1, Th2, and Treg cells, and a number of mouse models have shown that Th17 cells can promote CD8+ T-cell-mediated antitumor immune responses.^163^ In addition, the polarization of CD8+ T cells to Tc17 cells also increases antitumor immunity.^164^

#### CD8+ T cells

CD8+ T cells are cytotoxic T lymphocytes whose main role is to kill tumor cells directly. As mentioned previously, TAMs release immunosuppressive cytokines such as IL-10 and TGF-β, which directly inhibit the function of CD8+ T cells. Studies of the immunomodulatory function of TAMs have shown that they can inhibit CTLs and reduce their tumor-killing capacity in a number of ways.^165^ For example, the expression of PD-L1 and B7-1 (or CD80), which are ligands for the inhibitory checkpoint receptors PD-1 and CTLA4 expressed by activated T cells, leads to T-cell functionality by binding to PD-1 on CD8+ T cells “exhaustion”. Additionally, TAMs can reduce the bioavailability of L-arginine through the production of arginase 1 (ARG1), which is critical for T-cell function.^165–168^

#### Regulatory T cells/Tregs

CCL17 (also known as thymic and activation-regulated chemokine TARC) and CCL22 (also known as macrophage-derived chemokine MDC) are ligands for CCR4.^169–171^ The expression of CCL17 and CCL22 is elevated in breast cancer tumors^,^^172^ and the expression of CCL22 is increased in colorectal adenocarcinomas.^173^ CCL22 secreted by M2-type TAMs and other chemotactic factors can attract Tregs into the tumor microenvironment by attracting CCR4, the receptor for CCL22, to be highly expressed on Tregs; cytokines secreted by TAMs, such as IL-10 and TGF-β, can promote the proliferation and activation of Tregs; and at the same time, Tregs can in turn counteract macrophages, which tend to differentiate into M2-type macrophages, thus further supporting tumor growth. Recent findings have emphasized the integration of M2-polarized macrophages with immunostimulatory pathways to induce the differentiation of Treg cells.^174^

#### Natural killer T (NKT) cells

Natural killer T cells are a heterogeneous lymphoid population that may have both immune-enhancing and immune-suppressive effects, and in tumor immunity, the two NKT subpopulations (type I and type II) play opposing roles, which not only cross-modulate each other but also influence the innate immune cell population. In liver–lung metastasis models, type I NKT cells can rapidly release large amounts of Th1-type cytokines, such as IFN-γ, upon recognition of specific lipid antigens on the surface of tumor cells or macrophages.^175–178^ As mentioned previously, IFN-γ can induce polarization of TAMs toward the M1 type (antitumor phenotype) to enhance tumor immunity; CXCL16 is classified into a chemokine α subfamily and is induced by a variety of cells.^179^ CXCL16 is important for monocyte polarization to become macrophages in the tumor microenvironment, and when CXCL16 attracts monocytes, it is involved in recruiting them into the tumor ecotone, which then differentiates them into TAMs.^180,181^ Studies have shown that soluble CXCL16 (sCXCL16) may also be a macrophage-polarizing factor. sCXCL16 may also be a macrophage polarizing factor, and such polarized macrophages display characteristics of the M2 macrophage subpopulation: increased expression of CD163 and decreased expression of CD80, CD86, and HLA-DR. In addition, the secretion of large amounts of IL-10 and IL-15 by these macrophages also inhibits normal NK cell function.^182^

#### NK cells

The escape of tumor cells from immune surveillance is one of the key events regulating tumor growth, survival and metastasis. TAMs in an M2 macrophage-like state have poor antigen presentation capacity and suppress the immune response of T cells by releasing the immunosuppressive factors IL-10 and TGF-β, which include the inhibition of the cytotoxic function of NK cells.^183^ Kuang and colleagues reported that TNF-α and IL-10 secretion by activated monocytes strongly induced PD-L1 expression in an autocrine manner and that PD-L1-positive monocytes induced T-cell dysfunction, which was defined as low cytotoxicity against tumor cells and reduced T-cell proliferation.^184^ TAMs can bind to inhibitory receptors on NK cells, such as PD-1, through the expression of such inhibitory receptor ligands, which can in turn inhibit NK cell function.

### Dendritic cells (DCs)

The inflammatory nature of many cancers and the resulting tumor infiltration of various leukocytes (especially myeloid MDSCs and TAMs) combine to create an immunosuppressive environment that results in the suppression of the CD4+ and CD8+ T-cell response effects of DCs.^185,186^ This immunosuppression is usually mediated by cytokines secreted by tumor or tumor-infiltrating MDSCs and/or TAMs.^187^ For example, inhibitory cytokines secreted by TAMs, such as IL-10 and TGF-β, may inhibit the maturation of DCs, thereby reducing their antigen presentation capacity. Importantly, both MDSCs and TAMs in the tumor microenvironment can upregulate nitrogen oxide synthase expression and increase the production of NO and ROS, which affects the antigen-presenting function of DCs.^107^ Certain chemotactic factors and MMPs secreted by TAMs may also affect the migration of DCs. Owing to their properties, DCs are also known as “natural adjuvants”. It is used as a natural target for antigen delivery and acts as a bridge between the innate and adaptive immune responses, controlling tolerance and the immune response.^188^ IFN-γ and other cytokines secreted by DCs upon activation may promote the polarization of TAMs toward the M1 type (antitumorigenic type), and DC infiltration into tumors may also induce tumor growth and metastasis by modulating angiogenesis, host immunity, and tumor metastasis.^189^

Recent advancements in single-cell RNA sequencing have provided profound insights into the intricate interactions between TAMs and DCs within the TME. In hypopharyngeal squamous cell carcinoma (HPSCC), scRNA-seq analysis revealed a collaborative interplay between TAMs and LAMP3+ DCs, leading to the establishment of an immunosuppressive milieu that facilitates tumor progression by recruiting regulatory T cells while inhibiting CD8+ T-cell function. This interaction highlights the concerted efforts of TAMs and LAMP3+ DCs in promoting immune evasion mechanisms within tumors.^190^ Similarly, scRNA-seq profiling in early lung adenocarcinoma (LUAD) demonstrated an increased presence of both TAMs and CD1C+ DCs, which correlated with accelerated tumor progression. Although no distinct M1 or M2 polarization was observed, these cellular components likely contribute to immune evasion and tumorigenesis within the TME.^191^ These findings underscore the intricate interplay between TAMs and DCs in modulating both antitumor immunity and disease progression.

### Tumor-associated neutrophils (TANs)

TANs can secrete CXCL1, CXCL2, CXCL5, and other cytokines that may attract macrophages into the tumor microenvironment and affect their polarization.^192–195^ In parallel, chemotactic factors secreted by TAMs, such as CXCL8, may attract neutrophils into the tumor microenvironment.^196^ Studies involving the systemic inflammatory cascade of breast tumors triggered by IL-1β secretion by associated macrophages have shown that IL-17 expression by γδ T cells subsequently increases systemic granulocyte colony-stimulating factor (G-CSF) levels. Subsequent G-CSF stimulates neutrophil expansion and converts neutrophils into immunosuppressive cells, thereby blocking the antitumor function of CD8+ T cells and allowing disseminated cancer cells to evade immune detection.^197^

### B cells

There is also a close association between TAMs and B cells in the TME, where certain chemotactic factors secreted by TAMs may attract B cells into the TME and affect the balance of B-cell subsets. Cytokines (e.g., IL-6 and IgG) and immunomodulatory molecules produced by B cells may also affect the M1 and M2 polarization status of TAMs.^198^ Activated B cells can secrete chemokines, which increase the recruitment of TAMs to the tumor microenvironment and collectively influence T-cell responses, and can also promote tumor progression through degradation of the extracellular matrix and enhancement of angiogenesis in a granulocyte- and macrophage-dependent manner.^199^

### Kupffer cells

Tumor-associated macrophages and Kupffer cells are closely related in both physiological and pathological states. Kupffer cells, liver macrophages with M1 characteristics, can clear pathogens from the blood. Moreover, Kupffer cells exhibit the same phenotype, suggesting that TAMs and Kupffer cells undergo a dynamic transformation process in the tumor microenvironment.^56^ TAM receptors, particularly phosphatidylserine receptors such as Tim-4, are crucial for Kupffer cell phagocytosis of apoptotic cells. These receptors help Kupffer cells eliminate apoptotic cells and inflammatory mediators, reduce liver injury and inflammation, and modulate the inflammatory and immunomodulatory functions of Kupffer cells, which are crucial for liver health and disease treatment.^200,201^ Overall, the TAM–Kupffer cell interrelationship spans phagocytosis regulation, tumor microenvironment dynamics, and liver modulation.

## Role of antibody‒drug conjugates in TAM-based tumor therapy

In previous studies, monoclonal antibodies have been shown to be effective in the diagnosis and treatment of hematologic malignancies and various solid tumors,^202,203^ and they act by targeting tumor-associated antigens, which can inhibit cell growth and angiogenesis or stimulate a lasting immune response against tumors to achieve antitumor effects.^204,205^ ADCs have emerged as needed,^206^ which combines the targeting method of monoclonal antibodies with the ability of chemotherapy to kill tumor tissues while protecting healthy tissues, leading to major breakthroughs in the field of cancer treatment.^207,208^ To date, ADCs have become an important approach in cancer treatment. Infiltrating immune cells have been shown to play important roles in promoting tumorigenesis and progression,^93^ and TAM infiltration is usually associated with poor prognosis in cancer patients, which inevitably affects tumor therapy.^209^ TAMs are potent effectors of antibody-dependent cytotoxicity, contributing to the antitumor activity of anticancer monoclonal antibodies such as anti-CD20 and anti-HER-2.^210^ In fact, TAMs have been previously shown to be associated with the response to targeted anticancer drugs,^211^ and many studies have confirmed that trastuzumab can trigger the phagocytosis of human epidermal growth factor receptor 2 (HER2)-positive cells by macrophages,^212,213^ which suggests a potential role for TAMs in the antitumor activity of antibody therapy. The interaction between TAMs and ADCs is mediated by the Fcγ receptor (FcγR), which leads to the internalization of ADCs and treatment by TAMs, followed by the release of the payload in the TME.^214^ For further validation, Li et al. compared the antitumor activity and intratumoral drug concentration of targeted and nontargeted (hlgG-vcMMAE) monomethyl auristatin E (MMAE) conjugates and reported that nontargeted ADCs could bind to F4/80+TAMs, and their abundance correlated with the in vivo antitumor activity of nontargeted ADCs in lymphoma and breast cancer models. These findings demonstrated the ability of TAMs to internalize ADCs with FcγR and subsequently process ADCs to release their payload.^215^ Their study was the first to demonstrate this phenomenon even in the absence of antigen binding. TAMs can also interact with therapeutic ADCs. A study by Selby et al. demonstrated that anti-CTLA-4 antibodies act through macrophages expressing Fcγ receptors.^216^ They demonstrated in mouse model experiments that macrophage-mediated elimination of Treg cells by ADCC is an important component of anti-CTLA-4 therapeutic activity.^216^ Many previous studies have demonstrated that the development of ADC drugs that target TAMs may provide a new therapeutic approach for cancer treatment.

## Clinical applications and perspectives

### TAMs as biomarkers for prediction and intervention

TAMs, as important immune cells, can interact with various factors in the TME.^217^ As an increasing number of studies have explored the relationship between TAMs and tumors, we have shown that TAMs have unique characteristics during tumor progression to malignancy. Many studies have shown that TAMs in tumor tissues tend to polarize to the M2 type once they affect or interact with the tumor extracellular matrix,^218,219^ suggesting an important role for TAMs in early tumor prediction, therapeutic intervention and even prognosis prediction.

Jiao et al.^220^ The methylation and mRNA expression of Septin 9 (SEPT9) in different cervical tissues were detected via methylation-specific PCR and qRT‒PCR, which revealed that SEPT9 methylation promoted tumorigenesis and radioresistance in cervical cancer by targeting the HMGB1-RB axis and affected the resistance of cervical cancer to radiotherapy by mediating the ability of miR-375 to promote M2 polarization. These findings suggest that it may be a potential marker for early screening and intervention in patients with cervical cancer. Inagaki et al.^221^ used double immunofluorescence with CD68 and CD163 to evaluate the number, phenotype, and distribution of TAMs in 53 colorectal cancer (CRC) patients and reported that M2 macrophages increase with tumor progression, suggesting that M2 macrophages may play an important predictive role at the frontiers of tumor invasion, where the M2/M1 ratio is more predictive of lymphatic metastasis in CRC patients. Other related studies have also revealed a positive correlation between CD163 expression and the degree of lymphatic metastasis in either serum or CRC tissues,^222^ making it a novel biomarker with potential. Li et al. analyzed the cellular diversity and microenvironment heterogeneity of 91,394 single-cell transcriptomes from 18 clinical samples of non-atrophic gastritis (GS), intestinal metaplasia (IM), and GC patients and reported that TAMs exhibited a dominant M2-like phenotype, suggesting their immunosuppressive role in the tumor microenvironment and suggesting that TAMs may be potential predictors of GC.^223^

In recent years, targeted therapies for TAMs have focused on inhibiting the recruitment of TAMs, depleting TAMs, reprogramming TAMs into antitumor macrophages, and reversing the polarization of TAMs.^224^ The blockade of chemokines serves as a key to inhibiting the recruitment of TAMs, with CCL2/CCR2 being a popular target for recent studies. Using a mouse model, Yin et al. reported that stabilizing protein-1 interacting chitinase-like protein (SI-CLP) inhibits the cytoskeletal response to CCL2, alters the cellular composition of the TME, and ultimately prevents cytokine-induced recruitment of TAMs. Thus, it reduces macrophage infiltration in the mammary gland and achieves the effect of targeted therapy for breast cancer.^225^ In triple-negative breast cancer (TNBC), commonly used TNBC chemotherapeutic agents can activate TAMs and induce immune tolerance, which in turn affects the efficacy of chemotherapy.^226^ Plasticity is one of the key features of TAMs, which means that they can change their phenotype in the tumor microenvironment; therefore, reprogramming TAMs into antitumor macrophages is a very promising targeted therapeutic modality. Wang et al.^227^ reported that intravesical PA-MSHA (*Pseudomonas aeruginosa* mannose-sensitive hemagglutinin) treatment promoted an antitumor immune environment in a bladder cancer model characterized by an increase in mature TAMs, which indicated a shift toward M1-like macrophage polarization. Wang et al.^228^ investigated synthetic nanoparticles loaded with IL-12, which could functionally modulate TAMs for cancer immunotherapy. Umiker et al. developed a highly efficient and selective antagonistic monoclonal antibody (JTX-8064), which could be used to block the binding of leukocyte immunoglobulin-like receptor subfamily B member 2 (LILRB2) to its cognate ligand, thereby allowing human TAMs to be reprogrammed to drive T-cell activation in tumors to treat cancer.^229^

In addition, TAMs are closely related to tumor prognosis. Studies have shown that hypoxia is a typical feature of solid tumors,^230^ and in most solid tumors, the infiltration of high-density macrophages is correlated with poor prognosis. Wang et al. performed immunohistochemical staining of tumor tissues for TAMs and reported that patients with higher pathological grades of TNBC tended to have higher levels of TAMs, and their overall survival and disease-free survival were significantly shorter than those of patients with lower infiltration of TAMs.^231^ Among them, breast cancer patients with concomitant CD163+ and CD204 + TAM infiltrates tend to have a poor prognosis, as these TAMs are associated with rapid proliferation and poor differentiation.^232^ CD163, a specific tumor macrophage receptor, plays an important role in tumor progression. Ma et al.^233^ examined the expression levels of CD163 in patients with CRC versus healthy individuals, screened for four related genes and finally revealed that CD163 was differentially expressed in CRC tissues and was a poor prognostic factor. Yang et al.^234^ evaluated TAM markers (CD68 and CD163) in 81 CRC patients via immunohistochemistry and compared the survival rates of patients with high CD163+/CD68+ ratios with those of patients with low CD163+/CD68+ ratios. Patients with low ratios and patients with high CD163+/CD68+ ratios had a worse prognosis. However, Koelzer et al. analyzed 205 CRC patients in a study published in 2015 and reported that high CD163 + TAM infiltration implied a lower tumor grade and fewer lymphatic metastases, which predicted a better prognosis for CRC patients. Khaliq AM et al. performed droplet-based scRNA-seq on 16 racially diverse, treatment-naive CRC patient tissue samples and seven adjacent normal colonic tissue samples and found that the number of CAFs and C1Q+ TAMs was sufficient to stratify CRC patient prognosis with greater precision.^235^

### Discovery and application of CAR-Ms

Chimeric antigen receptor (CAR) T-cell therapy is not effective in solid tumor treatment, mainly because of the limited penetration and infiltration capacity of tumor cells, the presence of an immunosuppressive tumor microenvironment, and therapy-related adverse events such as targeted nontumor toxicity and cytokine release syndrome (CRS).^236^ To overcome the limitations of CAR-T cells in the treatment of solid tumors, researchers have explored the introduction of CAR technology into other innate immune cells, among which macrophages are an ideal choice because of their high proportion and versatility in the tumor microenvironment. In 2016, CAR-T-cell therapy specialists Saar Gill and Michael Klichinsky founded CARISMA Therapeutics, a company focused on developing CAR-macrophage therapies (CAR-Ms) for the treatment of tumors. In 2020, they published a research paper reporting that treatment with HER2-targeted CAR-M cells resulted in good tumor-killing effects in a mouse model and that it was able to transform M2 macrophages into M1 macrophages, induce an inflammatory TME, and enhance the antitumor cytotoxicity of T cells.^237^ Subsequent studies have shown that CAR-M cells have a significant therapeutic effect on a variety of different tumors. Two clinical trials based on CAR-M strategies have already been approved by the FDA. The first is CT-0508, a drug candidate from Carisma Therapeutics that treats patients with relapsed/refractory HER2-overexpressing tumors. On March 20, 2021, the Carisma team announced that it had completed the first patient administration of the phase 1 clinical study of CT-0508, which was the first CAR-M-cell therapy to enter the clinic, indicating that the new era of CAR-M-cell therapy officially opened. Another is MaxCyte’s drug candidate, MCY-M11, which uses Mesothelin-targeted CAR-M to treat patients with relapsed/refractory ovarian cancer and peritoneal mesothelioma and is currently recruiting volunteers for a phase I clinical trial.^238,239^ Another study constructed chimeric antigen receptor-macrophage (CAR-M) based on human peritoneal macrophage (PM) gene modification, namely, HF-CAR-PMS expressing HER2-Fc εR1-γ-CAR (HF-CAR). Through a variety of in vitro and in vivo gastric cancer model experiments, HF-CAR-PMs were found to specifically target HER2-expressing gastric cancer cells and trigger phagocytosis, significantly promoting HER2-positive tumor regression and prolonging overall survival in mouse models of peritoneal cancer, providing promising treatment options for HER2-positive gastric cancer patients.^240^ CAR-Ms have also shown significant potential in the treatment of brain tumors, with CAR-Ms targeting specific antigens such as HER2, EGFRvIII, IL-13Rα2, MSLN, B7-H3, and GPC-1 showing potent antitumor effects. Targeted therapy with HER2 and EGFRvIII has improved survival in preclinical models and is expected to breach the blood‒brain barrier, whereas IL-13Rα2 and MSLN have been shown to be potential targets for tumor clearance in brain tumors such as glioblastoma (GBM). Overall, CAR-M cells show promise in the treatment of brain tumors by targeting these key antigens.^241^ Although CAR-M cells have shown good results in clinical studies, they face several challenges in clinical application, including the high risk of gene transfer, the problem of cell origin is still unresolved, and the difficulty of delivering CARs in vivo. In addition, treatment limitations include a lack of tumor-specific antigens, low tumor invasion efficiency, and high mutation risk.^242^ However, the current research combined with nano, crisper and other technologies is expected to overcome this dilemma as soon as possible.^243,244^

### Future research directions and trends

TAMs are important components of the tumor microenvironment and account for a high percentage of immune cells. They are involved in the whole process of tumorigenesis, development and metastasis by promoting the growth of blood vessels and lymphatic vessels, inhibiting immune responses and regulating immune responses. In view of the important role of TAMs in tumor progression, TAM-based tumor prediction, prognostic assessment and targeted therapy have emerged. In the future, the construction of models based on TAM-related genes for the prediction of tumorigenesis and prognosis may be a very promising research field. In addition, research related to the cooperation of TAM-targeted therapy with immunotherapy, conventional chemotherapy and adjuvant therapy in the interventional treatment of patients with tumors is also a very promising option. Multimodal prediction and intervention based on TAM may become a hot research topic in the future.

## Strategies to regulate TAMs

**Fig. 5:**
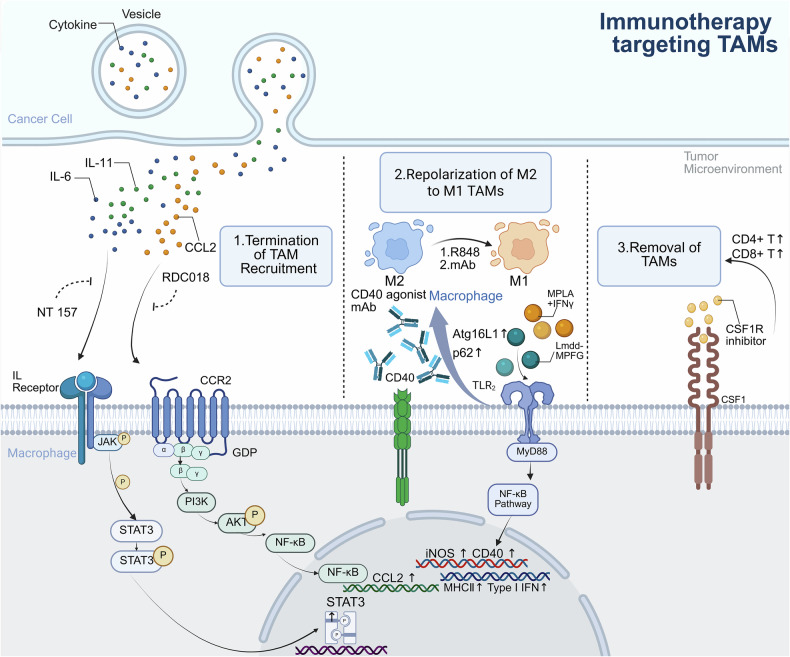
Interactions between tumor-associated macrophages and immune cells. Depiction of the complex interactions between TAMs and various immune cells, including T lymphocytes, NK cells, and dendritic cells. The figure explains how M1 and M2 TAMs influence the immune response through cytokine production, direct cell‒cell interactions, and modulation of the tumor microenvironment, ultimately affecting tumor progression and patient prognosis. (created with BioRender)

TAMs can contribute to tumor progression through a variety of pathways. For example, TAMs can promote the proliferation of fibroblasts and angiogenesis through high expression of proteases and achieve immunoprotection through the immunosuppression of T cells.^245^ In addition, studies have shown that TAMs are resistant to a variety of treatments and can impair the effects of various therapies, including immunotherapy, chemotherapy, radiotherapy and other therapeutic options.^246^ Therefore, it is necessary to modulate TAMs to improve patient prognosis and slow the progression of cancer. The general strategy of TAM regulation focuses on two aspects. First, regulating the number of TAMs, including reducing the recruitment of TAMs and eliminating local TAMs; second, altering the phenotype of TAMs and thus the function of TAMs, including re-educating the TAM phenotype to M1, weakens the tumor-promoting function of TAMs. (Fig. 5)

### Regulating the number of TAMs

A significant strategy of immunotherapy is regulating the number of TAMs. The following section will investigate this mechanism in detail. Table 2 summarizes the main target sites for TAMs regulation.

**Table 2:** Main target sites of immunotherapy in tumor-associated macrophage regulation

Pathways	Target	Drugs/Inhibitors	Change in TAMs	Refs.
Termination of Macrophage Recruitment				
CCL2/CCR2	CCR2	RDC018	RDC018 act as the CCR2 antagonist and reduces macrophage infiltration	^249^
		CCX872	Lower the ETV4-induced TAMs recruitment and infiltration, which further inhibits the HCC metastasis	^317^
		sc-202525 (CCR2 antagonist)	Reduced TAM infiltration	^252^
NF-κB/CCL2	CCL2	TGP	TGP reduces the release and recruitment of inflammatory factors, which suppress the infiltration of TAMs in TME	^318^
IGF-1R-IRS and JAK-STAT3	IGF-1R and STAT3	NT157	Short-term: No obvious change; Long-term: NT157 inhibited the production of TAM inducers, indicating a long-term inhibiting effect of TAM recruitment	^259^
CSF-1/CSF-1R	CSF-1R	BLZ945	Using a CSF-1R tyrosine kinase inhibitor (BLZ945) can counteract the improvement in total TAM content response to radiotherapy	^258^
Release of Immunostimulatory Capacity				
CCL2/CCR2	CCL2	LINC00330 (intergenic noncoding RNA)	The expression of LINC00330 is negatively linked to M2-type TAMs and positively linked to M1 TAMs. Reprogram TAMs polarization toward the M1 state.	^268^
NF-κB/CCL2	CCL2	TGP	TGP reduces the release of CCL2, which restrains TAM polarization toward the M2 state	^318^
CSF-1/CSF-1R	CSF-1R	M2pep-Modified Cyclodextrin-siRNA	Downregulation of CSF-1R achieved by siRNA reprogramming M2-type TAM into M1-type TAM	^287^
		CSF-1R-siRNA	Increase in M1 cytokine production and reduction in M2, which represent a repolarization of M2-type TAM into M1-type	^319^
		PLX3397 (CSF-1R inhibitor)	Impede M2-type TAM polarization and reprogrammed M2-type TAMs into M1-type	^316^
OX40/OX40L	OX40	OX40L M1-exos	Signature receptor CD86 of M1-type TAMs is upregulated, while CD206 for M2-type TAMs is downregulated.	^269^
OXCT1-Arg1	OXCT1	OXCT1 Knockout/Pimozide (OXCT1 inhibitor)	OXCT1 enriched in the M2-type TAMs and OXCT1 deficiency can induce the reprogramming of TAMs into tumor-suppressive type M1	^284^
Removal of TAMs				
CSF-1/CSF-1R	CSF-1R	BLZ945	Removal of TAMs in tissue specificity manner in cervical cancer and repolarization TAMs in microglia	^261,263^
		PLX3397	Depletion of TAMs and alter TAM-related gene expression toward M1 type	^264,265^

*ETV4* E-twenty-six-specific sequence variant 4, *HCC* hepatocellular carcinoma, *TAMs* tumor-associated fibroblasts, *TGP* total glucosides of paeony

#### Termination of macrophage recruitment

Clinical experiments have confirmed that TAMs can help tumor cells form an inflammatory environment after tumorigenesis, which is conducive to the growth of tumor cells, while their ability to promote angiogenesis enhances the migration and invasion ability of tumor cells.^247^ Tumor cells are able to secrete some cytokines to recruit TAMs through their cellular pathways of action, further constructing a tumor microenvironment that is conducive to the growth of tumor cells and contributes to tumor development.^248^ Therefore, termination of macrophage recruitment in cancer tissues can block macrophage growth and signaling pathways of differentiation, thus reducing the ability of TAMs to promote tumor development and spread.

##### CCL2/CCR2 signaling pathway

The CCR2 antagonist RDC018 can target and block the CCL2/CCR2 signaling pathway, thereby inhibiting TAM migration and aggregation. An examination of mice with in situ hepatocellular carcinoma after administration of the CCR2-blocking drug revealed a notable reduction in the clustering of TAMs. As a result, the interaction between TAMs and tumor cells decreased. Additionally, M2-type TAMs show a decrease in the production of cytokines and chemokines, while there is a trend toward an increase in the number of CD8+ and CD4+ T cells, which have antitumor effects.^249^ These phenomena suggest that CCR2 antagonists are able to reduce the infiltration of intratumorally M2-type TAMs and increase the number of CD8+ T cells and CD8+ TILs with tumor-killing effects in the microenvironment of peripheral blood. Small molecules capable of influencing gene expression along this pathway can indirectly impact TAM recruitment. For example, glioblastoma cells produce kynurenine, which can activate AHR in TAMs. AHR has been demonstrated to exhibit high expression and activity in multiple cancer types.^250^ Furthermore, AHR enhances CCR2 expression, thereby promoting TAM recruitment.^251^ Additionally, the TLR9-mediated NF-κB signaling pathway is activated during cytosolic mitochondrial DNA (mtDNA) stress, leading to the production of CCL2. This molecule facilitates TAM recruitment and is associated with hepatocellular carcinoma (HCC) progression, indicating a close interconnection between signaling pathways.^252^

Several drugs have been demonstrated to have ancillary effects on TAM recruitment. Total glucosides of paeony (TGPs) have been found to inhibit the release of inflammatory factors, and there is evidence of their anti-inflammatory and immunomodulatory effects.^253,254^ Jin et al.^255^ revealed the great role and therapeutic potential of TGP in altering the inflammatory microenvironment of tumors, revealing that TGP can be used to inhibit the release of inflammatory factors via the NF-κB/CCL2 signaling pathway and thus reduce the recruitment of TAMs in the tumor microenvironment, as well as to inhibit M2-type polarization by decreasing the expression of mRNAs in lipopolysaccharide-stimulated macrophages (LPS-stimulated macrophages), ultimately exerting a regulatory effect on TAMs. A reduction in the release of inflammation-related factors (e.g., CCL2) further reduces the infiltration of TAMs. It improves the inflammatory microenvironment of the tumor, ultimately inhibiting tumor growth and metastatic processes.

##### CSF-1/CSF-1R signaling pathway

Targeting the CSF-1/CSF-1R signaling pathway, a pathway closely associated with TAM proliferation and differentiation, has also been shown to effectively halt TAM recruitment. Several macrophage-targeting agents targeting this pathway are currently under clinical evaluation.^256^ In glioma patients undergoing radiotherapy, TAM accumulation has been observed posttreatment in mice. TAMs can interfere with therapeutic outcomes by modulating interactions between tumor and stromal cells.^257^ Leila Akkari et al. reported an increase in total TAM populations in glioblastoma-bearing mice treated with ionizing radiation (IR), which was inhibited by the CSF-1R tyrosine kinase inhibitor BLZ945. Notably, administering BLZ945 post-IR treatment did not significantly affect total TAM numbers, as pretreatment with BLZ945 prevented IR-induced TAM population changes. Post-IR administration of BLZ945 reversed only TAM-related transcriptional signatures induced by IR therapy.^258^

##### Other signaling pathways

NT157, a small-molecule inhibitor of immunotherapeutic drugs, can exert its antitumor effects mainly through two signaling pathways, the IGF-1R-IRS and JAK-STAT3 pathways. Sanchez-Lopez et al.^259^ found that NT157 reduces the aggregation of TAMs in tumor cells, mainly by blocking the JAK-STAT3 signaling pathway. Cytokines such as IL-6, which has the ability to protect intestinal epithelial cells from apoptosis, are inducible to STAT3. By blocking the expression of these cytokines, NT157 can block the activation of STAT3, which further achieves the goal of inhibiting the recruitment of TAMs and reducing the number of TAMs.

#### Removal of TAMs

The expression of cytokines within tumors promotes the proliferation of TAMs. For example, the signaling pathway hosted by CSF-1/CSF1R plays an essential role in the recruitment of TAMs and the promotion of polarization of TAMs toward the M2 type.^260^ CSF1R inhibitors promote the depletion of M2-type TAMs in a tissue-specific manner, revealing the potential of CSF1R as a therapeutic target for cancer treatment. Strachan et al.^261^ investigated a small molecule inhibitor of CSF1R, BLZ945, and found that tumor volume was reduced in the model treated with BLZ945. Further experiments revealed that BLZ945 exhibits tissue specificity in the clearance of TAMs, with incomplete clearance of macrophages from neoplastic lesions and the surrounding cervical stroma observed in a cervical cancer model after treatment with BLZ945. Similar results were obtained in the breast. Notably, in a mouse model of microglia,^262^ instead of removing TAMs, BLZ945 inhibited the polarization of TAMs toward the M2 phenotype, thereby affecting tumor progression. These findings suggest that the effect of BLZ945 on TAMs is tissue specific. Another CSF1R inhibitor, pexidartinib **(**PLX3397), also has a depleting effect on the removal of TAMs upon acceptance and dose-dependently downregulates the expression of genes related to polarization toward M2 and upregulates the expression of genes related to polarization toward M1, which is similar to the action of BLZ94.^263,264^

In addition, both BLZ945 and PLX3397 were able to modulate the immune cell infiltration profile. For example, increasing the immune infiltration of CD8+ T cells into tumor sites. In combination therapy with monoclonal antibodies, this can further promote the polarization of TAMs toward the M1 type.^265^ These findings suggest that CSF1/CSF1R is a viable therapeutic target and may be used in combination with other therapies. These discoveries provide promising therapeutic approaches and treatment ideas for immunotherapy.

### Release of immunostimulatory capacity

One way to regulate TAMs is by increasing their immunostimulatory capacity. TAMs can be polarized into M1-type and M2-type TAMs, where M1-type TAMs have antitumor and immune-promoting effects,^266^ whereas M2-type TAMs promote tumor development by suppressing the immune system. In recent years, M2-type TAMs have also been shown to be strongly associated with weaker immunotherapeutic efficacy and other adverse clinical outcomes, such as drug resistance in patients during anti-PD-1/PD-L1 immunotherapy.^267^ Therefore, the activation of M1-type TAMs with antitumor activity, or the promotion of TAM polarization to the antitumor type, can enhance the ability of TAMs to phagocytose and kill tumor cells. Reeducation of M2 TAMs can effectively achieve this goal. Reprogramming TAMs to release their immunostimulatory capacity could be achieved via three main strategies: signaling pathway regulation, cytokine regulation and metabolite regulation, which reverse the phenotype of TAMs to remodel the tumor microenvironment.

#### Signaling pathway regulation

##### CCL2/CCR2 signaling pathway

The CCL2/CCR2 pathway is a critical regulator of TAMs. When the intergenic noncoding RNA LINC00330 specifically binds to CCL2, it acts as an inhibitor of CCL2/CCR2 and its downstream factors through autocrine signaling. This interaction mediates TAM reprogramming, promoting the polarization of M2-type TAMs to M1-type TAMs.^268^

##### OX40/OX40L signaling pathway

OX40L, a molecule expressed on macrophages, participates in the OX40/OX40L signaling pathway. OX40L-overexpressing M1-like macrophage exosomes derived from M1-like macrophages overexpressing OX40L can bind to OX40-expressing T cells and activate this pathway. Coculture of these exosomes with M2 TAMs in vitro resulted in a reduction in the M2 marker CD206 and a significant increase in the M1 marker CD86, indicating that M2-type TAMs are reeducated into M1-type TAMs via the IFN-γ secreted by CD8+ T cells. This finding highlights the critical role of cytokines in reprogramming TAM polarization.^269^

##### The CD47-SIRPα signaling pathway

Tumor cells evade macrophage phagocytosis by overexpressing CD47, which binds to SIRP-α on macrophages, transmitting a “do not eat me” signal. Consequently, the CD47-SIRPα axis is now recognized as an immune checkpoint for macrophages and a potential target for immunotherapy. Research has shown that blocking the CD47-SIRPα signaling pathway could actively shift TAMs toward an antitumor profile by reeducating the M2 type and enriching the M1 type to restore phagocytic function. Tang et al.^270^ reported M1-macrophage-derived hybrid nanovesicles (SPI@hEL-RS17) with RS17, a CD47-specific antitumor peptide that effectively inhibits CD47-SIRPα signaling. Blockade of CD47 enabled M1 TAMs to be enriched in the TME and produce greater tumor-phagocytosing effects. Similar effects were observed in Zhao’s research, which constructed a stimuli-responsive multifunctional nanoplatform (ZIF-PQ-PDA-AUN) to increase the phagocytotic ability of macrophages, reversing M2-type TAMs into M1-type TAMs.^271^ Although direct mechanistic evidence for TAM re-education is limited, these findings, which succeeded in reversing the TAM phenotype, provide a feasible and promising strategy for improving the therapeutic index with low-toxicity immunotherapy.

##### Other signaling pathways

TAMs can participate in antibody-dependent cellular phagocytosis (ADCP) by mediating the phagocytosis pathway and eliminating target cells through specific antibodies. Li et al.^272^ reported that resiquimod (R848) promotes ADCP by stimulating M1-type macrophages and shifting TAMs from the M2 phenotype to the M1 phenotype. Compared with monotherapy, combination therapy with TLR7/8 agonists resulted in more efficient TAM re-education. Figueiredo et al.^273^ M2-type TAMs were targeted with mUNO peptides on lignin nanoparticles (LNPs) carrying a dual agonist of the Toll-like receptor TLR7/8 (Resiquimod, R848), achieving effective low-dose treatment and re-educating M2-type macrophages to M1-type macrophages, enhancing the antitumor immune response.

The Wnt signaling pathway was demonstrated to be a driver of the immunosuppressive phenotype of M2 TAMs, which could also affect the communication between tumor cells and TAMs.^274^ Thus, drugs that affect Wnt signaling may exert an indirect effect on TAMs. Andrographolide (ADE), a drug that can directly promote the apoptosis of tumor cells through cytotoxicity and inhibit tumor proliferation and metastasis, has recently been demonstrated to play an important role in regulating TAMs. Li et al.^275^ reported that ADEs significantly inhibited the polarization of TAMs toward the M2 phenotype and promoted the polarization of the M1 phenotype, ultimately inhibiting triple-negative breast cancer. Further research on the underlying mechanism by transcriptome sequencing revealed that ADEs act mainly on the Wnt signaling pathway, inhibiting the expression of the Wnt5a, β-catenin, MMP-9 and MMP-2 proteins in the signaling pathway in a dose-dependent manner, thus reducing the invasion, metastasis and angiogenesis of tumor cells.

#### Cytokine regulation

Cytokine intervention can influence TAM polarization and offer new immunotherapy possibilities.^260^. Sun et al.^276^ found that the combination of monophosphoryl lipid A (MPLA) and interferon-gamma increased the expression of M1 markers (iNOS and CD40) and decreased the expression of an M2 marker (CD206), indicating TAM polarization toward the M1 phenotype. This therapy alters the immune microenvironment by secreting chemokines, increasing oxidative stress in tumor cells, and stimulating MHC class II gene transcription, thus killing tumor cells. Ahirwar et al.^277^ found that Slit2, a tumor suppressor, could regulate TAMs in the breast cancer tumor microenvironment, including increasing the recruitment of M1-type TAMs and increasing their phagocytosis of tumor cells, which demonstrated that Slit2, a tumor suppressor, could effectively play an antitumor role and revealed the potential of Slit2 as an immunotherapeutic agent. Metformin was reported to be able to reeducate the M2 type to the M1 type. Wei et al.^278^ used mannose-modified macrophage-derived microparticles (Man-MPs), which exhibit a stronger repolarization ability than free metformin, to load metformin. This is achieved through the AMPK‒NF-κB signaling pathway, which regulates the expression of M1- and M2-type cytokines.^279^

#### Metabolite regulation

As mentioned above, evidence demonstrating the potent effects of small metabolic molecules on the CCL2/CCR2 pathways highlights their ability to modulate TAM recruitment, underscoring the intricate crosstalk between metabolic pathways and TAMs. The polarization state of TAMs can reprogram metabolic processes, thereby influencing disease progression.^280,281^ Conversely, metabolic changes within the body can also affect TAM polarization.^282,283^ This concept is supported by evidence across various biological processes. 3-Oxoacid CoA-transferase 1 (OXCT1), a key enzyme in ketolysis, has been identified as a regulator of TAM polarization. Elevated OXCT1 expression in TAMs promotes the production of succinate, a byproduct with TAM-reprogramming capabilities, which functions via the H3K4me3-Arg1 axis. Notably, in OXCT1mKO mice, OXCT1 deficiency in TAMs does not affect their proliferation, indicating that OXCT1 has no significant effect on the overall TAM population. However, inhibition or deletion of OXCT1 activity, for example, via the use of pimozide (PZ), can reprogram M2-type TAMs into the M1 phenotype, thereby enhancing antitumor immunity.^283,284^

### Strategies for regulating TAMs via gene editing techniques

#### CRISPR/Cas9 system to regulate TAMs

**Fig. 6:**
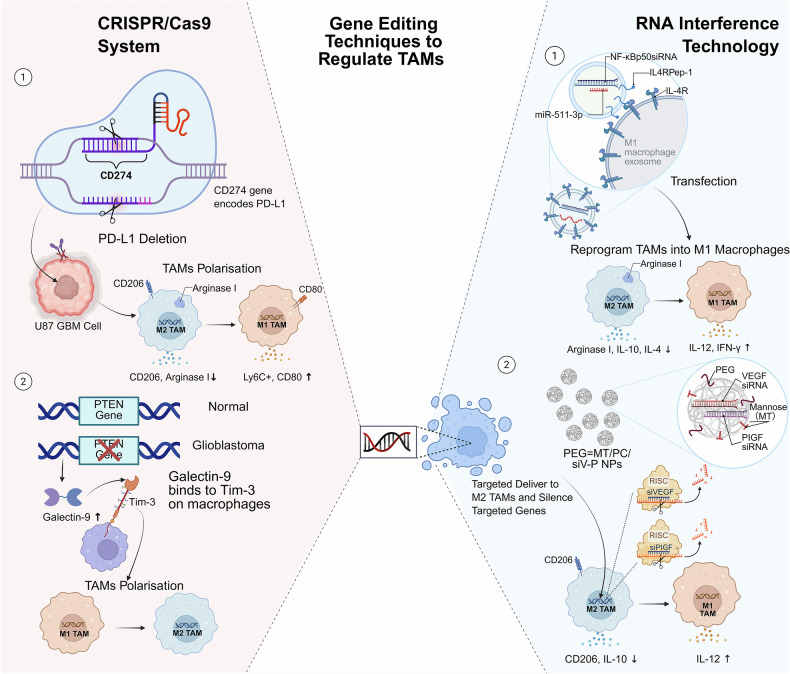
Therapeutic strategies targeting tumor-associated macrophages. This figure outlines various therapeutic approaches aimed at modulating TAM activity, including immunotherapies, pharmacological interventions, and gene editing techniques. This study highlights strategies such as re-education of M2 TAMs to the M1 phenotype, inhibition of TAM recruitment, and depletion of TAM populations, highlighting their potential. (created with BioRender)

CRISPR/Cas9 is a commonly used gene editing technology that enables the editing and regulation of specific genes in TAMs, allowing for precise alteration of their phenotype and function.^285^ Zhao et al.^286^ used bacterial protoplast-derived nanovesicles to deliver CRISPR-Cas9, which targets Pik3cg in TAMs. This suppressed PI3Kγ signaling, reducing the expression of M2 markers (CD206 and IL-10) and increasing the expression of M1 markers (CD86 and TNF-α), which inhibited tumor growth by reprogramming TAMs in 4T1 and MC38 mouse models. The success of this practice provides a viable option to achieve the regulation of TAMs by effectively silencing genes that promote TAMs. In glioblastoma, a tumor suppressor gene, the phosphatase and tensin homolog (PTEN) gene, is deleted, leading to increased secretion of galectin-9 (Gal-9), which induces polarization of TAMs toward the M2 type. Knockdown of the CCR2 gene effectively reduces the level of infiltration of TAMs and achieves the clearance of TAMs.^262^ (Fig. 6).

#### RNA interference technology

Targeted gene silencing technology mediated by siRNA or shRNA is able to inhibit or reduce the expression of target genes, thus altering the phenotype or function of TAMs and regulating them. Targeting the CSF-1/CSF-1R signaling pathway to disrupt CSF-1R expression has demonstrated significant therapeutic potential across various cancers. In prostate cancer (PCa), a study utilizing M2 macrophage-targeting peptides for the delivery of siRNA specifically to M2 TAMs reported an increase in M1 TAM expression, accompanied by a decrease in the expression of M2-specific markers (CD68+/CD206+). These findings indicate the successful reprogramming of M2 TAMs into the M1 phenotype via siRNA-mediated modulation.^287^ Similarly, in pancreatic cancer (PC), siRNA-mediated interference targeting the same pathway in combination with the PI3K-γ inhibitor BEZ235 yielded comparable outcomes. Notably, BEZ235 alone demonstrated the ability to modulate TAM phenotypes through the suppression of the phosphorylated AKT (pAKT) pathway. Moreover, the combination therapy exhibited synergistic effects, significantly enhancing TAM phenotype reprogramming compared with single agent treatments, with markedly reduced production of IL-6, a signature cytokine of M2 macrophages that is also closely associated with pancreatic cancer progression. In experiments where IL4RPep-1 was modified on the surface for targeted transfection of NF-κBp50 siRNA as well as miR-511-3p into M1-type exosomes, significant downregulation of the expression of M2 markers and cytokines (Arg1, TFG-β, IL-10, and IL-4) was observed, whereas M1 cytokines (IL-12p40 and IFN-γ) were upregulated, suggesting the ability to reprogram the M2 type to the M1 type.^288^ Researchers have designed nanoparticles (NPs), which include trimethyl chitosan (PEG = MT) and citraconic anhydride-grafted poly(allylamine hydrochloride) (PC) (PEG = MT/PC NPs). The NPs were then double modified with polyethylene glycol (PEG) and mannose (MT) and internally wrapped with VEGF siRNA (siVEGF)/PIGF siRNA (siPIGF) for targeted delivery to M2-type TAMs. These NPs then aggregated at high concentrations in the tumor cells and effectively silenced the target genes after their contents were released. This ultimately repolarizes the M2 type to the M1 type and achieves the regulation of TAMs.^289^ Selective silencing of genes related to M2-type TAMs can effectively reduce the aggregation of M2-type TAMs. However, to achieve this goal, the targeting ability and effectiveness of siRNA transfer into target cells need to be improved, and the combination of nanotechnology may be a promising solution.

### FDA-approved drugs and clinical studies based on TAM regulation

Macrophages exhibit dual protective and pathogenic effects on cancer development. As macrophages, especially TAMs, are essential immune cell components in the TME, the distribution of TAMs is closely associated with cancer progression, therapeutic response and prognosis. As mentioned above, proper modulation of macrophages can increase their antitumor ability. Thus, unraveling the antitumor potency of M1 macrophages through several regulatory strategies to leverage their phagocytic function has now become the main strategy in cancer therapy and has already been proven in several preclinical and clinical studies. The FDA has approved several macrophage-based therapies, and more drugs are now in the clinical trial stage.

#### CD47-SIRPα signaling pathway

Employing the strategies discussed earlier in the CD47-SIRPα signaling pathway could reveal the potential of identifying and phagocytosing macrophages. Several drugs that block this pathway have been tested for therapeutic efficacy and toxicity in clinical trials (Table 3).

**Table 3:** Clinical trials of drugs targeting the CD47-SIRPα signaling pathway to regulate macrophages

Drug	Clinical Trials ID	Phase	Enrollment	Other therapy	Indications	Clinical Outcome	TEAEs	Refs.
Anti-CD47 Monoclonal Antibody								
CC‑90002	NCT02641002^a^	I	28	\	R/R AML and MDS	\	SAE: 82%; TRAE: 32–46%	^320^
Magrolimab	NCT02953782	Ib/II	78	With Cetuximab	Advanced Solid Malignancy and CRC	PFS: KRASwt CRC: 3.6 months KRASm CRC: 1.9 months OS: KRASwt CRC: 9.5 months KRASm CRC: 7.6 months;	SAE^b^: 25–60% TRAE: 5%	^321^
	NCT03558139	I	34	With Avelumab	Checkpoint-Inhibitor-Naive Ovarian Cancer Patients	PFS: 2.0 months OS: 10.2 months	SAE: 0–66.67%	^322^
Lemzoparlimab (TJC4)	NCT04202003	I/IIa	105	\	R/R AML and MDS	1 patient in primary refractory AML achieved the morphologic leukemia-free state	TRAE: 40%	^323^
Ligufalimab (AK117)	NCT04349969	I	15	With or without Cadonilimab (AK104)	Advanced or Metastatic Solid Tumors or Lymphomas	\	TRAE: 10.5% (5 cases)	^324^
	NCT04728334	I	49	\	Advanced Solid Tumors or Lymphomas	\	\	^325^
Anti-SIRPα Signaling Pathways								
BI-765063	NCT04653142	I	17	With BI-754091	Advanced Solid Tumors	\	SAE: 41%; TRAE: 53%	^326^
	NCT03990233	I	50	\	Advanced Solid Tumors	In HCC: PR: 0.02%	TRAE: mild to moderate	^327^
Anzurstobart (CC-95251)	NCT03783403	I	17	With Rituximab	R/R Non-Hodgkin Lymphoma	ORR: 41%;	TRAE: 29%	^328^
BI 770371	NCT05327946	I	36	With or without Ezabenlimab	Advanced Solid Tumors	\	\	^329^

Target	Drug	Clinical Trials ID	Phase	Enrollment	Other therapy	Indications	Clinical Outcome	TEAEs	Ref.
Bispecific CD47-targeting antibodies (bsAbs)									
CD47/ PD-L1	IBI322	NCT04328831 NCT04912466	Ia/Ib	58	\	Advanced Malignant Tumors	PR: 20% SD: 35%	74.1%	^330^
		NCT04795128^b^	I	24	\	Anti-PD-(L)1 Treatment-Resistant Classic Hodgkin lymphoma	ORR: 47.8%; DCR: 91.3%	91.7%	^331^
	HX009	NCT04097769	I	21	\	Advanced Malignancies	PR: 14.2%; SD: 28.6%	SAE: 4.7%; TRAE: 47.6%	^332^
CD47/CD20	IMM0306	NCT05805943	I	48	\	R/R CD20-positive B-cell Non-Hodgkin’s Lymphoma	PFS: 10.58 months;	SAE: 16.7% Grade ≥3 TRAE: 68.8%	^333^
		NCT05771883	I	8	With Lenalidomide	R/R CD20-positive B-cell Non-Hodgkin’s Lymphoma	ORR: 71.4%; DCR: 85.7%	SAE: 12.5% (1/8 Grade ≥3 TRAE: 75.0%	^334^
CD47/ CD19	TG-1801	NCT03804996	I	30	With or without Ublituximab	R/R B-cell Lymphoma	Monotherapy: PR: 21.4% Combination: ORR: 44%; PR: 37.5%	Monotherapy: TRAE: 30.0% Combination: TRAE: 31%	^335^
	NI-1701	NCT04806035^a^	Ib	21	With or without Ublituximab	B-cell Lymphoma or Chronic Lymphocytic Leukemia	\	\	^296^
CD47 /HER2	IMM2902	NCT05805956^a^	I/II	105	\	HER2-expressing Advanced Solid Tumors	\	\	^336^
		NCT05076591^a^	I	135	\	HER2-expressing Advanced Solid Tumors	\	\	^337^
CD47/ 4-IBB	DSP107	NCT04440735	I	23	\	Advanced Solid Tumors	SD: 50%	Grade 1-2 TRAE: 70%	^338^
SlRPα-Fc Fusion Protein									
SIRPα-Fc-CD40L	SL-172154	NCT04406623^a^	I	27	\	Platinum-Resistant Ovarian Cancer	SD: 22%	Grade ≥3 TRAE: 14%	^339^
		NCT04502888^a^	I	5	\	Squamous Cell Carcinoma of the Head and Neck or Skin	\	\	^340^
		NCT05483933^a^	Ib	86	With Mirvetuximab or PLD	Ovarian Cancers	\	\	^341^
SIRPα-Fc lgG1	IMMO1	ChiCTR 1900024904	I	14	\	R/R Lymphoma	SD: 7.1%	SAE: 7.1%	^342^
	ALX148	NCT03013218	I/II	41	With or without chemotherapy or immunotherapy	Advanced Head and Neck Squamous Cell Carcinoma (HNSCC) or HER2-positive GC	HNSCC: ORR: 38.5% GC: ORR: 72.2%	HNSCC: 15.4% GC: 44.4%	^307^
	TTI-621	NCT04996004	I/II	23	With or without Doxorubicin	Unresectable or Metastatic High-grade Leiomyosarcoma	PR: 25.0% SD: 55%	Monotherapy: Grade ≥3 TRAE: 9.0% Combination: Grade ≥3 TRAE: 30.0%	^343^
SIRPα-FclgG4	TTI-622	NCT05261490	I/II	10	With Pegylated Liposomal Doxorubicin	Platinum-Resistant Ovarian Cancer	SD: 50%	SAE: 10%	^344^
		NCT03530683	I	42	\	Advanced Hematologic Malignancies (Lymphoma, Leukemia and Multiple Myeloma)	ORR: 33.3%	TRAE: 47%	^345,346^

*AML* acute myeloid leukemia, *CRC* colorectal cancer, *DCR* disease control rate, *GC* gastric/gastroesophageal cancer, *HER2* human epidermal growth factor receptor 2, *HNSCC* Head and Neck Squamous Cell Carcinoma, *ORR* objective response rate, *OS* overall survival, *MDS* Myelodysplastic Syndromes, *PFS* progression-free survival, *PLD* pegylated liposomal doxorubicin, *PR* partial response, *R/R* relapsed and/or refractory, *SAE* Serious Adverse Events, *SD* stable disease, *TEAEs* Treatment-Emergent Adverse Events

^a^Clinical trials have been terminated, suspended or are still in the recruiting stage

^b^The data presented in the table are from a dose-expansion cohort study

##### Monoclonal antibodies

Immunotherapy uses humanized IgG4 anti-CD47 antibodies (Abs) or anti-SIRPα antibodies. First-generation Abs, including CC-90002 and Magrolimab, exhibit off-target effects, which lead to relatively severe hematotoxicity. Moreover, the next-generation Abs Lemzoparlimab and Ligufalimab reduce off-target binding to red blood cells (RBCs), which does not cause substantial hematotoxicity. Both generations block CD47, which has been shown to enhance antitumor effects on hematological malignancies and solid tumors. These drugs are currently in clinical trials, with promising outcomes in the next-generation group.

The first generation of anti-CD47 antibodies appears to have a high incidence of off-target adverse effects. This is because CD47 is widely expressed in somatic cells, especially red blood cells (RBCs), which cause adverse hematologic events, including anemia and thrombocytopenia, and hematotoxicity seems to be Fc dependent. Several clinical trials have been terminated because of intolerable treatment-related adverse effects (TRAEs).^290–292^ With respect to the Fc portion of anti-CD47 monoclonal antibodies (mAbs), researchers have reported that the Fc-FcγR interaction is required to fully trigger macrophage phagocytosis and maximize antitumor effects.^293^ However, current anti-CD47 mAbs are mainly humanized IgG4, which lacks Fc-FcγR interaction. This finding reveals the need for restructuring the Fc portion to achieve AE alleviation and increased therapeutic efficacy.

Next-generation antibodies are targeted to minimize off-target effects by reducing the binding of anti-CD47 Abs to RBCs and platelets. Compared with previous anti-CD47 mAbs, significant alleviation of AEs was observed in clinical trials. Patients with CD20-positive non-Hodgkin’s lymphoma in the relapsed and refractory (R/R) state have a 100% disease control rate (DCR) with manageable TRAEs, mainly Grade 1 or 2 TRAEs, after receiving Lemzoparlimab.^294^ In addition, the strong tumor-targeting effect indicates the effectiveness of RBC-sparing therapy. This provides a new strategy for R/R patients and reveals the potential of differentiated Abs.

##### Bispecific targeting antibodies

Anti-CD47 mAbs exhibit limited therapeutic efficacy when combined with other therapies. This limitation arises because, compared with conventional monoclonal antibodies that bind to a single antigen, bispecific antibodies (bsAbs) can engage two distinct epitopes on the same cell, increasing their affinity and activating or restraining diverse signaling pathways.^295^ This also helps shape the TME into an antitumorigenic state with alterations in immune cell infiltration and immune-related gene regulation.^296^ The use of bsAbs enables Fc–FcγR interactions, which traditional monoclonal antibodies’ IgG portion cannot achieve, thereby activating key biological processes such as antibody-dependent cell-mediated cytotoxicity (ADCC) and antibody-dependent cellular phagocytosis (ADCP). These processes significantly improve the efficacy of both monotherapy and combination therapy, particularly in tumors in which multiple ligands are expressed.^297^ Furthermore, the design of bsAbs with low CD47 affinity can reduce hematotoxicity, addressing one of the major side effects associated with CD47 targeting.^298^ Current co-targeting strategies with CD47, including PD-L1, CD20, CD19, HER2, and 4-IBB (CD137), are being actively investigated in multiple clinical trials. Moreover, additional targets, including CD24, CD33, CD38, CD70, CD123, EGFR, and receptor glypican-3 (GPC3), are under investigation in preclinical studies. For signal-regulatory protein alpha (SIRPα), co-targeting with 4-1BB is also being studied, highlighting the promising future of bsAbs in oncology, which has shown effective repolarization of the TAM phenotype, promotion of phagocytosis, and a potent ability to enhance immunotherapeutic effects^299–307^ (Table 3).

#### CSF-1/CSF-1R signaling pathway

As inhibiting CSF-1R could block the CSF-1/CSF-1R signaling pathway, which is responsible for macrophage differentiation and proliferation, re-education of TAMs could lead to therapeutic therapy for advanced tumors.^308,309^ The FDA approved PLX3397 (pexidartinib), a CSF-1R inhibitor, in 2019 as the first and only drug to target CSF-1R for the treatment of malignant fibrous histiocytoma (TGCT), with more drugs being in clinical trials. PLX3397 inhibition of CSF-1R selectively impacts the viability and polarization processes of M2 macrophages, which also enhances cytotoxic CD8+ T-cell infiltration, thereby achieving a tumor-suppressive microenvironment by halting the tumorigenic effects of crosstalk between tumors and M2 TAMs without interfering with the antitumor effects of M1 macrophages.^310,311^ Preclinical evidence has shown that this preferentiality could enhance the therapeutic effects of other treatments, supporting the promising role of CSF-1/CSF-1R in cooperation with other therapeutic drugs and its ability as a prognostic factor.^310,312^ However, the hepatic toxicity of PLX3397 cannot be ignored and may be induced by the depletion of resident macrophage Kupffer cells in the liver, resulting in impaired physiological enzymatic clearance.^313,314^ Strategies targeting CSF-1/CSF-1R have focused mainly on CSF-1R inhibitors, including small molecules and antibodies, while the latter seem to be more likely to have immune-related adverse effects.^315^

The current systematic administration of PLX3397 may cause off-target adverse effects, which introduces the need for targeted delivery. Nanotechnology enables precise drug delivery, enhances therapeutic outcomes and reduces off-target effects. Codelivery of CSF-1R inhibitors with other agents or cytokines via nanocarriers can further reshape the immune microenvironment and strengthen antitumor immunity. Hu et al.^316^ used nanotechnology to co-deliver IL-12-expressing genes and the CSF-1R inhibitor PLX3397, effectively activating T-cell-mediated immunity and reducing the number of M2-type TAMs more significantly than single-drug delivery. This approach induced substantial changes in the tumor microenvironment, demonstrating superior efficacy in impeding M2 polarization.

## Conclusion

Tumor-associated macrophages (TAMs), which act as both allies and adversaries during cancer progression, are central players in the tumor microenvironment. This review synthesizes decades of research to unravel their complex biology, clinical relevance, and therapeutic potential. TAMs, derived from circulating monocytes or tissue-resident macrophages, are highly adaptive, polarizing into different functional states—inflammatory M1 or immunosuppressive M2 phenotypes—according to signals from their surroundings. M1 TAMs fight tumors through phagocytosis and cytokine release, and M1 TAMs fight tumors through phagocytosis, cytokine release, and immune activation, whereas M2 TAMs drive tumor growth, angiogenesis, and immune evasion. However, this M1/M2 dichotomy is oversimplified. Advances in single-cell technology have led to the discovery of multiple TAM subpopulations, such as thrombospondin-1+ (THBS-1+) macrophages in hepatocellular carcinoma and FN1+ TAMs in gliomas, each with unique transcriptional profiles and clinical significance. These findings challenge traditional classification methods and emphasize the need for a nuanced understanding of TAM heterogeneity.

The dual role of TAMs is governed by complex signaling pathways. For example, the NF-κB and STAT3 pathways drive protumorigenic functions such as angiogenesis and immunosuppression, whereas the CSF-1/CSF-1R axis regulates TAM recruitment and survival. The CD47-SIRPα signaling pathway, a key “do not-eat-me” signal utilized by tumors, has emerged as a key therapeutic target. Blocking CD47 enhances phagocytosis by macrophages, a strategy currently being tested in clinical trials. Macrophages also interact dynamically with other immune cells: M2 TAMs recruit regulatory T (Treg) cells via CCL22, suppress CD8+ T cells via PD-L1, and inhibit NK cell activity, thus collectively creating an immunosuppressive environment. Conversely, reprogramming M2 TAMs to an M1-like state via cytokines such as IFN-γ or TLR agonists restores antitumor immunity, highlighting their plasticity and therapeutic potential.

In the clinical setting, TAM represents both a burden and an opportunity. High M2 TAM infiltration is associated with poor prognosis in cancers such as breast, colorectal and pancreatic cancer, making it a valuable biomarker. For example, CD163+ TAMs are predictive of shorter survival in triple-negative breast cancer patients, while spatial distribution patterns provide additional prognostic insights. Therapeutic strategies targeting TAMs aim to eliminate TAMs, block their recruitment, or reprogram their function. CSF-1R inhibitors such as PLX3397 reduce the number of immunosuppressive M2 TAMs in preclinical models, whereas anti-CD47 antibodies disrupt “do not-eat-me” signaling to promote phagocytosis. Combining these approaches with chemotherapy or immunotherapy holds promise for overcoming resistance.

Emerging therapies such as CAR-M cells represent a paradigm shift. By modifying macrophages to express chimeric antigen receptors, researchers have created cells capable of selectively targeting tumors, such as HER2+ cancers, while reprogramming the TME. Early trials, such as those involving CT-0508, which targets HER2-positive solid tumors, demonstrated its feasibility and antitumor activity. Similarly, nanotechnology-driven delivery systems can precisely reprogram TAMs in situ, minimizing off-target effects. These innovations emphasize the potential of TAM-focused therapies to complement existing therapies.

However, significant challenges remain. The M1/M2 framework, while useful, fails to capture the full scope of TAM diversity observed in human tumors. Single-cell studies have revealed environmentally relevant subpopulations that cannot be simply categorized; thus, multidimensional classification systems are needed. For example, Kupffer cell-like TAMs in hepatocellular carcinoma differ functionally from microglia-derived TAMs in glioblastoma, reflecting tissue-specific adaptations. In addition, therapeutic strategies that are successful in mice tend to fail in humans because of compensatory mechanisms. CSF-1R inhibitors, although effective at depleting M2 TAMs in preclinical models, have limited clinical efficacy as monotherapies, in part because tumors recruit alternative myeloid cell populations to maintain immunosuppression. The metabolic flexibility of TAMs complicates targeted therapies. M1 TAMs rely on glycolysis, whereas M2 TAMs favor oxidative phosphorylation, a dichotomy that is affected by metabolites such as succinate and itaconic acid. Drugs targeting metabolic enzymes can reprogram TAMs by altering metabolite levels, but their effects vary by tumor type. Similarly, organelle-specific interventions, such as modulating mitochondrial fission in TAMs, show preclinical promise but require deeper mechanistic validation.

Future research must prioritize several areas. First, integrating multi-omics data—spatial transcriptomics, proteomics, and metabolomics—will shed light on how TAM subpopulations interact with other cells, such as cancer-associated fibroblasts and endothelial cells, to form TMEs. Second, the development of tailored therapies is critical. For example, TAMs in early-stage tumors may require different targeting strategies than TAMs in metastatic niches. Third, improved preclinical models—using patient-derived organ tissues or humanized mice—will improve translational relevance. Finally, the use of artificial intelligence to analyze TAM heterogeneity and predict treatment response could accelerate the development of personalized medicine.

In summary, TAMs are double-edged swords in cancer biology. Their ability to inhibit or promote tumors depends on environmental cues, making them dynamic therapeutic targets. While current strategies, such as CSF-1R inhibition, CD47 blockade, and CAR-M, are promising, overcoming resistance and environment-dependent variability requires innovative approaches. By embracing the complexity of TAM biology and advancing technologies such as single-cell analysis and nanotechnology, novel therapies that transform the TME from a barrier to a battleground for immune-mediated tumor eradication can be developed. This progress will depend on interdisciplinary collaborations that bridge the gap between immunology, bioengineering, and clinical oncology to provide lasting solutions for cancer patients.
